# Adenylate Cyclase AcyA Regulates Development, Aflatoxin Biosynthesis and Fungal Virulence in *Aspergillus flavus*

**DOI:** 10.3389/fcimb.2016.00190

**Published:** 2016-12-21

**Authors:** Kunlong Yang, Qiuping Qin, Yinghang Liu, Limei Zhang, Linlin Liang, Huahui Lan, Chihao Chen, Yunchao You, Feng Zhang, Shihua Wang

**Affiliations:** Fujian Key Laboratory of Pathogenic Fungi and Mycotoxins, Key Laboratory of Biopesticide and Chemical Biology of Education Ministry, and School of Life Sciences, Fujian Agriculture and Forestry UniversityFuzhou, China

**Keywords:** adenylate cyclase, sclerotia, cAMP, *Aspergillus flavus*, aflatoxin

## Abstract

*Aspergillus flavus* is one of the most important opportunistic pathogens of crops and animals. The carcinogenic mycotoxin, aflatoxins produced by this pathogen cause a health problem to human and animals. Since cyclic AMP signaling controls a range of physiological processes, like fungal development and infection when responding to extracellular stimuli in fungal pathogens, in this study, we investigated the function of adenylate cyclase, a core component of cAMP signaling, in aflatoxins biosynthesis and virulence on plant seeds in *A*. *flavus*. A gene replacement strategy was used to generate the deletion mutant of *acyA* that encodes the adenylate cyclase. Severe defects in fungal growth, sporulation and sclerotia formation were observed in the *acyA* deletion mutant. The defect in radical growth could be partially rescued by exogenous cAMP analog. The *acyA* mutant was also significantly reduced in aflatoxins production and virulence. Similar to the former studies in other fungi, The *acyA* mutant showed enhancing tolerance to oxidative stress, but more sensitive to heat stress. Overall, the pleiotropic defects of the *acyA* deletion mutant indicates that the cAMP-PKA pathway is involved in fungal development, aflatoxins biosynthesis and plant seed invasion in *A. flavus*.

## Introduction

*Aspergillus flavus* is a saprophytic soilborne fungus that contaminates food-stuffs and a broad range of important agricultural crops, including maize, peanut, and cottonseed, with the most carcinogenic metabolite, aflatoxins (AFs) (Amaike and Keller, 2011; Yang et al., 2015). This fungus is also one of the most opportunistic pathogen of human and animals causing aspergillosis diseases or liver cancer either through consumption of contaminated food or through invasive growth (Hedayati et al., 2007; Amaike and Keller, 2011; Yang et al., 2015). Because of this, *A. flavus* causes food shortages, significant economic losses and health problems all over the world especially in warm and moist fields (Amaike and Keller, 2011; Bai et al., 2015). Effective strategies of combating this pathogen are required to alleviate its potential deleterious effects.

The cAMP/PKA signaling pathway, which utilizes cyclic AMP (cAMP) as a second messenger, controls a range of physiological processes in eukaryotic cell (Oliver et al., 2002; Xue et al., 2008). In fungi, cAMP regulates both metabolism and morphogenesis (Thevelein and de Winde, 1999; Lengeler et al., 2000; Fillinger et al., 2002). As study in *S. cerevisiae*, cAMP regulates carbon metabolism, cell cycle progression, and pseudohyphal growth (Ward et al., 1995). In filamentous fungi, such as *N. crassa*, cAMP signaling is also required for hyphal tip growth, conidiation, and carbon metabolism (Kays et al., 2000). In many plant-pathogenic fungi, cAMP signaling is also involved in toxin production and virulence (D'Souza and Heitman, 2001; Lee et al., 2003; Shimizu et al., 2003; Choi and Xu, 2010; Hu et al., 2014). For example, in *F. graminearum*, cAMP signaling has been shown to be important for the production of DON, which is one of the best characterized virulence factors (Hu et al., 2014; Jiang et al., 2016). In *A*. *nidulans*, cAMP/PKA pathway is also known to negatively regulate sterigmatocystin biosynthesis (Shimizu et al., 2003).

In the cAMP network, the intracellular cAMP level is synthesized by the core component, adenylyl cyclase, when responding to extracellular stimuli. Adenylate cyclase has been studied for decade, which is shown to have pleiotropic effects on growth, conidiation, sexual development, and virulence in phytopathogenic fungi (Terenzi et al., 1979; Adachi and Hamer, 1998; Choi and Xu, 2010). Although adenylate cyclase plays various roles in a wide range of fungi, the function of adenylate cyclase in secondary metabolism and infection in *Aspergillus* has yet been investigated. In this study, we are interested in revealing the roles of adenylate cyclase in modulating the cAMP levels and signaling during different stages of development and AF biosynthesis in *A. flavus*. Disruption of cAMP signaling by deletion of the *A. flavus* adenylyl cyclase gene has pleiotropic effects on fungal development, conidiation and stress responses. We also provide data on the impact of cAMP-signaling on aflatoxin biosynthesis and virulence in *A. flavus*.

## Materials and methods

### Strain and culture conditions

*Aspergillus flavus* wild-type (WT) strain and all the transformants in this study (Table 1) were cultured on potato dextrose agar (PDA) for mycelia growth assays. For determining sensitivities to various stresses, fungal growth was assayed after incubation at 37°C for 4 d on PDA plates with 200 μg/mL Congo red, 100 μg/mL Calcofluor white (CFW), 100 μg/mL sodium dodecyl sulfate (SDS), 20 mM/L hydrogen peroxide (H_2_O_2_), 2.0 mM/L tert-butyl hydroperoxide (tBooH), 1M NaCl and 1M KCl. To analyse conidia production, 1 × 10^3^ *A. flavus* conidia was pointed onto PDA agar media, then grown for 5 d at 37°C under dark conditions. Three 1.5 cm diameter cores were harvested from the center of each plate and homogenized in 3 mL of distilled water, and the spore number was counted haemocytometrically. For sclerotial production analysis, sclerotial inducing WKM medium was used (Chang et al., 2012). Cultures were grown at 37°C for 7 d under dark condition, and the plates were then sprayed with 70% ethanol to kill and wash away conidia to aid in enumeration of sclerotial. The experiment was conducted with technical triplicates for each strain, and was repeated thrice.

**Table 1:** **Wild-type and mutant strains of fungi used in this study**.

**Strain**	**Genotype description**	**References**
*A. flavus* PTS	Δ*ku70*; Δ*niaD*; Δ*pyrG*	Chang et al., 2010
wild-type	Δ*ku70*; Δ*niaD*; Δ*pyrG*::*pyrG*	This study
*ΔacyA*	Δ*ku70*; Δ*niaD*; Δ*pyrG*; *ΔacyA*::*pyrG*	This study
*ΔacyA-C*	Δ*ku70*; Δ*niaD*; Δ*pyrG*; *ΔacyA*::*pyrG*; *acyA* (*p*):: *acyA*::*ptrA*	This study
G*acyA*	Δ*ku70*; Δ*niaD*; Δ*pyrG; acyA(p)*::eGFP-AcyA::*pyrG*	This study

### Targeted gene deletion and complementation

To generate the *acyA* deletion strain (Δ*acyA*) and the Δ*acyA* complementary strain (Δ*acyA-C*) of *A. flavus*, a previously described approach was used (Yang et al., 2016). The primers used in this study to amplify the fragment for gene knockout were listed in Table 2. The fusion PCR approach was used to generate gene replacement constructs for the *acyA* gene, and the fusion PCR product was transformed into protoplasts of the *A. flavus* PTS strain. For complementation, *acyA* ORF with its native promoter was amplified using primer pairs CM-*acyA*/F and CM-*acyA*/R (Table 2), and then cloned into the pPTR I vector (Takara). The recombinant plasmid, harboring *acyA* ORF with its native promoter and a pyrithiamine-resistance marker, was transformed into protoplasts of the Δ*acyA* mutant. Preparation of protoplasts and fungal transformation were performed as previously described (Cary et al., 2006; Abdel-Hadi et al., 2011; Yang et al., 2016).

**Table 2:** **Gene-specific primers used for gene knock-out**.

**Primers**	**Sequence(5′–3′)**	**Application**
*acyA*/P1	TCACCATTCCGACCGACAG	*acyA* deletion and probe
*acyA*/P3	GGGTGAAGAGCATTGTTTGAGGCTGTCAAACGAGGAAGAGCAC	
*acyA*/P4	GCATCAGTGCCTCCTCTCAGACGTTGGATGTTCAGCGTTCAG	
*acyA*/P6	CCAGTTACCGTTGAGACCG	
*acyA*/P2	ATGAGGGTGAGGTCCATTCT	
*acyA*/P5	CCTCCAGAATCCGTATGAGC	
PyrG/F	GCCTCAAACAATGCTCTTCACCC	*acyA* deletion
PyrG/R	GTCTGAGAGGAGGCACTGATGC	
P801/R	CAGGAGTTCTCGGGTTGTCG	
*acyA*-ORF/F	GCGAAAGCGGACAGGTAAGA	*acyA* mutant screen
*acyA*-ORF/R	GCCTGGTTCATCTGGTCCTA	
CM-*acyA*/F	CTATGACCATGATTACGCCAAGCTTGACAGGAGCCAAAGAAGTAGG	*acyA* complementation construct
CM-*acyA*/R	CCAGTGAATTCGAGCTCGGTACCGGTGTGTCAGGTCGCCAGTT	
eGFP-pyrG/F	GGAGCTGGTGCAGGCGCTGGAGCCGGTGCCATGGTGAGCAAGGGCGAGGA	*egfp*
eGFP-pyrG/R	GGGTGAAGAGCATTGTTTGAGGCTTACTTGTACAGCTCGTCCATG	
*acyA*-eGFP/P1	GGCTGCCTTGCTATGGTGT	*acyA-gfp* tag construct
*acyA*-eGFP/P3	GGCTCCAGCGCCTGCACCAGCTCCTGGGATGCTCATGCGTAGAA	
*acyA*-eGFP/P2	CTTCGTGTCCTCGGATTTCA	

### Seed infections

The ability of the wild-type and all the mutant strains to infect crop seeds was assayed as described previously (Tsitsigiannis and Keller, 2006; Kale et al., 2008; Yang et al., 2016). The peanut cotyledons treated with *A. flavus* conidia were incubated at 29°C for 5 d at dark conditions, and the filter paper was moistened daily. After incubation for 5 d, the peanut and maize seeds were harvested in 50 mL Falcon tubes, weighed, then with vortex for 2 min to release the spores in 15 mL of sterile water supplemented with 0.05% Tween 80. Conidiation was counted haemocytometrically. There were three replicated plates per strain and the experiment was repeated thrice.

### Aflatoxins analysis

To analyse aflatoxin (AF) production, 10 mL aliquot of a 10^6^ spore/mL suspension of *A. flavus* conidia was incubated into Glucose Minimal Media(GMM) (Shimizu and Keller, 2001) medium in the dark at 29°C for 5 d. AF extraction was followed as previously described (Yang et al., 2016). Thin Layer Chromatography (TLC) was used to analyse AF production, performed as previously described (Yang et al., 2016). The experiment was conducted with technical triplicates for each strain, and was repeated thrice.

### Quantitative RT-PCR

For qRT-PCR, the mycelia of wild-type and all the mutant strains were harvested at growth stages (48 h and 72 h incubated on PDA). Total RNA was isolated with TRIzol reagent (Biomarker Technologies, Beijing, China), and the first-strand cDNA was synthesized with All-in-One First-Strand cDNA Synthesis SuperMix (TransGen Biotech, Beijing, China). QRT-PCR was performed with the Thermo Fisher Scientific Real-time PCR System (Finland) using TransStart Top Green qPCR SuperMix (TransGen Biotech, Beijing, China). In the quantitative real-time PCR, AF structural gene *aflD* and regulator gene *aflR* were amplified by the primer pairs shown in Table 3, and *actin* gene was used as the endogenous reference gene. The relative quantification of each transcript was calculated following the 2^−ΔΔCT^ method (Livak and Schmittgen, 2001). All qRT-PCR assays were conducted with technical triplicates for each sample, and the experiment was repeated three times.

**Table 3:** **Gene-specific primers used for RT-PCR**.

**Primers**	**Sequence(5′–3′)**	**Application**
*brlA/QF*	GCCTCCAGCGTCAACCTTC	*brlA* qRT-PCR
*brlA/QR*	TCTCTTCAAATGCTCTTGCCTC	
*abaA/QF*	CACGGAAATCGCCAAAGAC	*abaA* qRT-PCR
*abaA/QR*	TGCCGGAATTGCCAAAG	
*nsdC*/QF	GCCAGACTTGCCAATCAC	*nsdC* qRT-PCR
*nsdC*/QR	CATCCACCTTGCCCTTTA	
*nsdD*/QF	GGACTTGCGGGTCGTGCTA	*nsdD* qRT-PCR
*nsdD*/QR	AGAACGCTGGGTCTGGTGC	
*laeA*/QF	TTGTTGGGGTTGACCTTGCT	*laeA* qRT-PCR
*laeA*/QR	GCCATCCCATCACACTTCCA	
*aflD*/QF	GTGGTGGTTGCCAATGCG	*aflD* qRT-PCR
*aflD*/QR	CTGAAACAGTAGGACGGGAGC	
*aflR*/QF	AAAGCACCCTGTCTTCCCTAAC	*aflR* qRT-PCR
*aflR*/QR	GAAGAGGTGGGTCAGTGTTTGTAG	
*aflO*/QF	GATTGGGATGTGGTCATGCGATT	*aflO* qRT-PCR
*aflO*/QR	GCCTGGGTCCGAAGAATGC	
*acyA*/QF	AAACGGCCCCAAGACAGTGG	*acyA* qRT-PCR
*acyA*/QR	AGCCCGGGTATAGCCTCTCG	
Actin/QF	ACGGTGTCGTCACAAACTGG	*actin* qRT-PCR
Actin/QR	CGGTTGGACTTAGGGTTGATAG	

### Intracellular cAMP measurement

Cultures of 2-day-old liquid mycelial were harvested, frozen in liquid nitrogen and lyophilized more than 6 h. Intracellular cAMP extraction was followed as previously described (Liu et al., 2007). The cAMP levels were quantified according to the Direct cAMP colorimetric (EIA) kit (Enzo Life Sciences, Exeter, UK). The experiment was conducted with technical triplicates for each strain, and was repeated thrice.

### Tagging of AcyA with eGFP under the native promoter

To localize AcyA, a eGFP-pyrG fragment was amplified from pKNTG vector using primer pairs GFP-pyrG-F/ eGFP-pyrG-R (Table 2). A same approach was used to construct the AcyA-GFP fusion cassette as described previously (Zheng et al., 2012; Yang et al., 2016). In brief, a 1.0 kb fragment immediately upstream of the *acyA* stop codon and a 1.0 kb fragment immediately downstream of the *acyA* stop codon were amplified from wild-type strain using primer pairs *acyA*-eGFP/P1/*acyA*-eGFP/P3 and *acyA*/P4/*acyA*/P6, respectively. The AcyA-eGFP fusion PCR cassettes (using primer pairs *acyA*-eGFP/P2 and *acyA*-eGFP/P5) were transformed into *A. flavus* PTS protoplast, and the transformants embedding homologous integration were verified by PCR.

### Microscopic analysis

Bright field and epifluorescence microscopy was the Olympus BX51 microscope (Olympus, Japan) equipped with a ×20 0.5 NA (numerical aperture), ×40 1.3 NA, or ×100 1.40 NA Olympus oil immersion objective lens. Alternatively, confocal microscopy was used for time-lapse or live cell fluorescence imaging using a Nikon A1R laser scanning confocal microscope system (Nikon, Japan). GFP excitation was performed with 488 nm light (Em. 525/40 nm).

### Statistical analysis

All data were presented as means± standard deviation of at least three biological replicates samples. Statistical and significance analysis were performed using the GraphPad Prism 6 and regarded significant if *p*-values were < 0.05. Student's *t*-test was used when comparing two means for differences. For multiple comparisons, Tukey's multiple comparison test was used for significance analysis.

## Results

### Identification and analysis of adenylate cyclase in *A. flavus*

**Figure 1:**
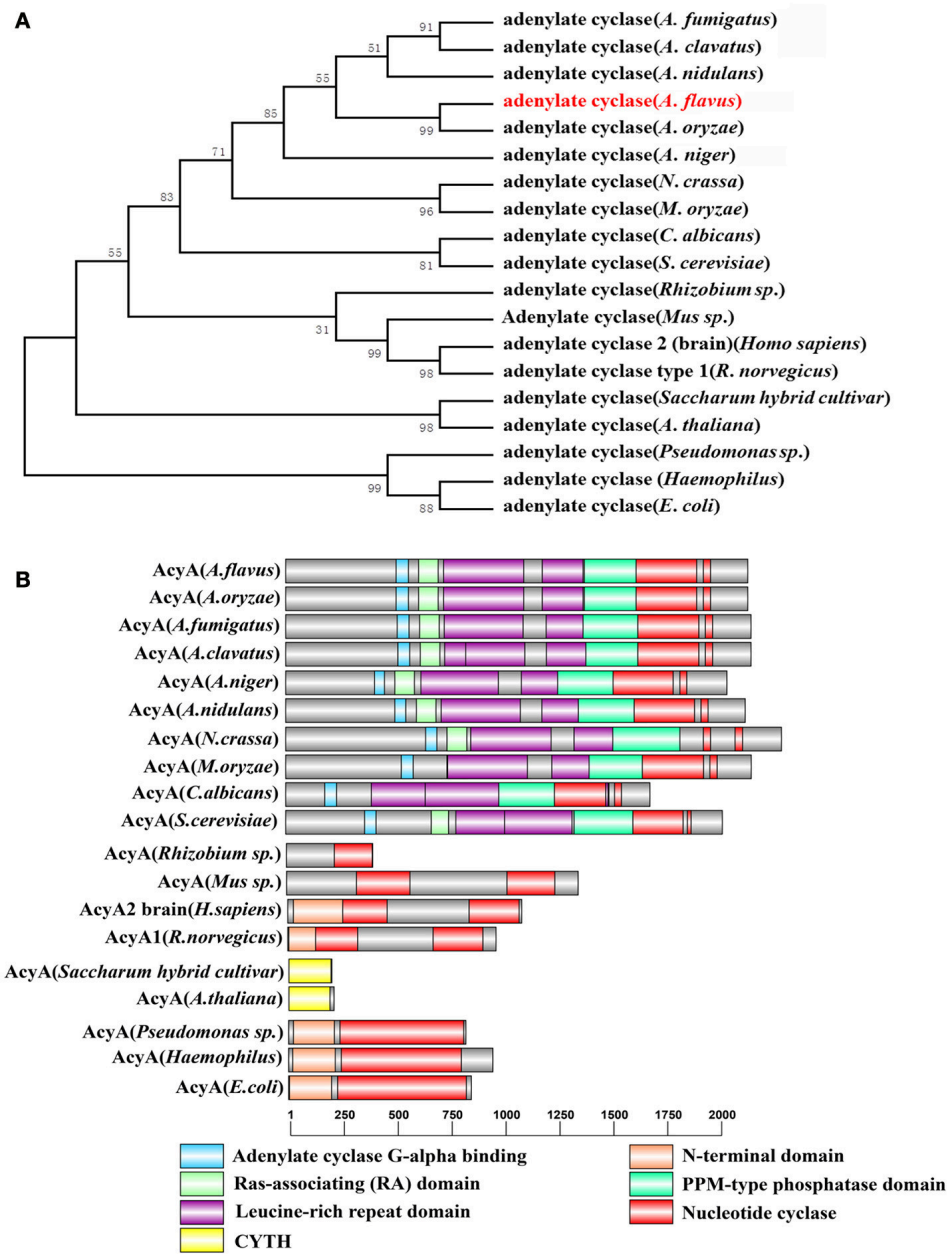
**Identification of adenylate cyclase in ***A. flavus***. (A)** Phylogenetic analysis of adenylate cyclase. The phylogenetic tree based on all the available adenylate cyclase amino acid sequences from different organisms was constructed by MEGA5.0 software using the Neighbour-joining method. Bootstrap analysis was performed with 1000 replicates. **(B)** Sequence structure of AcyA was identified by SMART (http://smart.embl-heidelberg.de/smart/set_mode.cgi?NORMAL=1), and the domain architectures were visualized using software DOG 2.0.

cAMP/PKA pathway is one of the most important signaling pathways in eukaryotic organisms. To identify ortholog of the yeast adenylate cyclase in *A. flavus*, the protein sequences of yeast CYR1 (AAA34549.1) was used as queries for BlastP analyses in the NCBI using the Basic Local Alignment Search Tool (http://blast.ncbi.nlm.nih.gov/Blast.cgi). AFLA_128130 in *A. flavus* was predicted to encode a 2144 aa protein, showing 35% overall identity to yeast CYR1, which we designated herein as AcyA. A phylogenetic analysis of the evolutionary relationship of the AcyA in *Aspergillus, Magnaporche. oryzae, Neurospora. crassa, Candida albicans, Saccharomvces cerevisiae, Pseudomonas sp*., *Mus sp*., *Homo sapiens, Rattus norvegicus, Saccharum hybrid cultivar, Arabidopsis thaliana, Pseudomonas sp., Haemophilus, and Escherichia coli* (Figure 1A) revealed that AcyA were conserved among *Aspergillus*. The amino acid sequence alignment of adenylate cyclase in different fungi shows that AcyA contains several highly conserved domains (Figure S1), from the N- to C-terminus, including an Adenylate cyclase G-alpha binding domain, Ras-associating (RA) domain, Leucine-rich repeat domain, PPM-type phosphatase domain and Nucleotide cyclase, however, in bacteria or animals the adenylate cyclase only have two domains, the N-terminal domain and Nucleotide cyclase(Figure 1B).

### Location of AcyA

**Figure 2:**
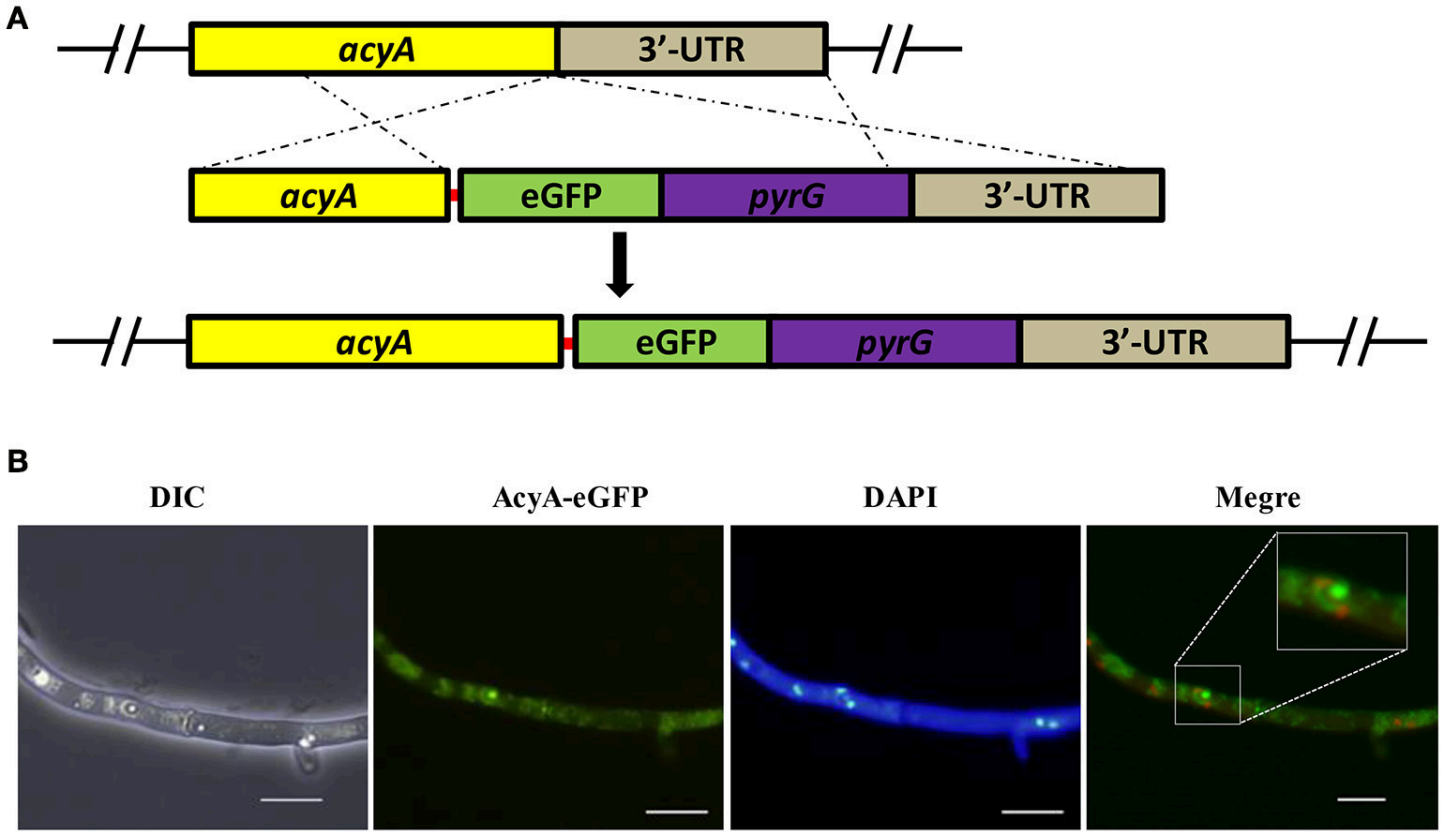
**Subcellular distribution of AcyA-eGFP fusion protein. (A)** Diagrammatic representation of the gene replacement strategy for construction of *acyA(p)-acyA*-e*gfp* strain. The red line represents a 5 Gly–Ala linker between AcyA and eGFP to make sure these two proteins can function independently. **(B)** Localization of AcyA-eGFP in the vegetative growth period, mycelia were collected from YES media after grown at 37°C for 24 h.

In order to track the subcellular localization of AcyA, we generated eGFP tag at the C-terminus of AcyA protein under the control of theri native promoter. The AcyA::eGFP transformants showed a WT phenotype (data not shown), indicating that the AcyA-GFP fusion is fully functional. The strategy for their construction is illustrated in Figure 2A. As shown in Figure 2B, in the vegetative growth period, it showed a strong fluorescence signal in the cytoplasm and nucleus of the hyphae.

### AcyA is essential for hyphal growth and conidiation

**Figure 3:**
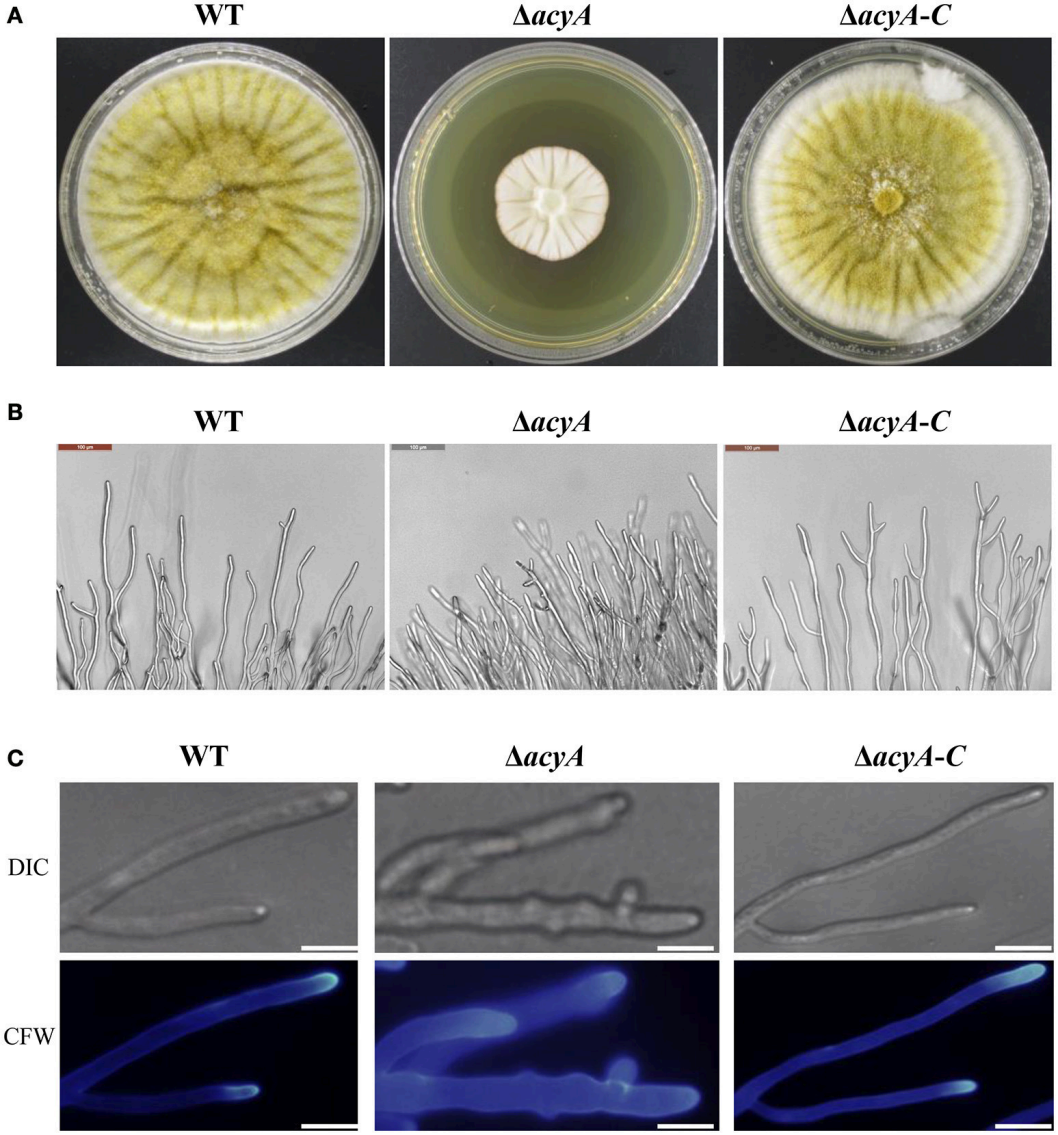
**The Δ***acyA*** mutant was defective in colony morphology. (A)** Colonies formed by the wild type, Δ*acyA* and Δ*acyA-C* complemented strain on YES agar plates. **(B)** Microscopic examination revealed the difference mycelia tips of WT, Δ*acyA* and Δ*acyA-C* complemented strain, bars = 100 μm. **(C)** mycelia of WT, Δ*acyA* and Δ*acyA-C* complemented strain were stained with Calcofluor white (CFW) and examined by DIC or fluorescence microscopy, bars = 200 μm.

To gain insight into the function of adenylate cyclase during growth and morphogenesis in *A. flavus*, we generated gene-deletion mutants of *acyA* (Δ*acyA*) in the PTS wild-type strain. PEG-mediated gene targeting was used to replace the entire ORF of the *acyA* gene with the *pyrG* selective marker (Figure S2A). The selected transformants were confirmed to be knockouts by PCR, and the failure of gene expression in Δ*acyA* was also verified by RT-PCR (Figure S2B). Furthermore, we constructed a complementation strain (Δ*acyA-C*) by reintroducing the genomic DNA sequence encompassing *acyA* ORF and 1 kb promoter region. When grown on YES agar medium for 4 d at 37°C, the Δ*acyA* mutant was significantly reduced in growth rate compared to the wild-type and Δ*acyA-C* strain (Figure 3A). Colonies formed by the Δ*acyA* mutant also had limited aerial hyphal growth. Further microscopic examination showed that Δ*acyA* mutant tended to produce substantial hyperbranching (Figures 3B,C, Figure S3). These results indicated that the AcyA plays an important role in the vegetative growth and hyphal branching of *A*. *flavus*.

**Figure 4:**
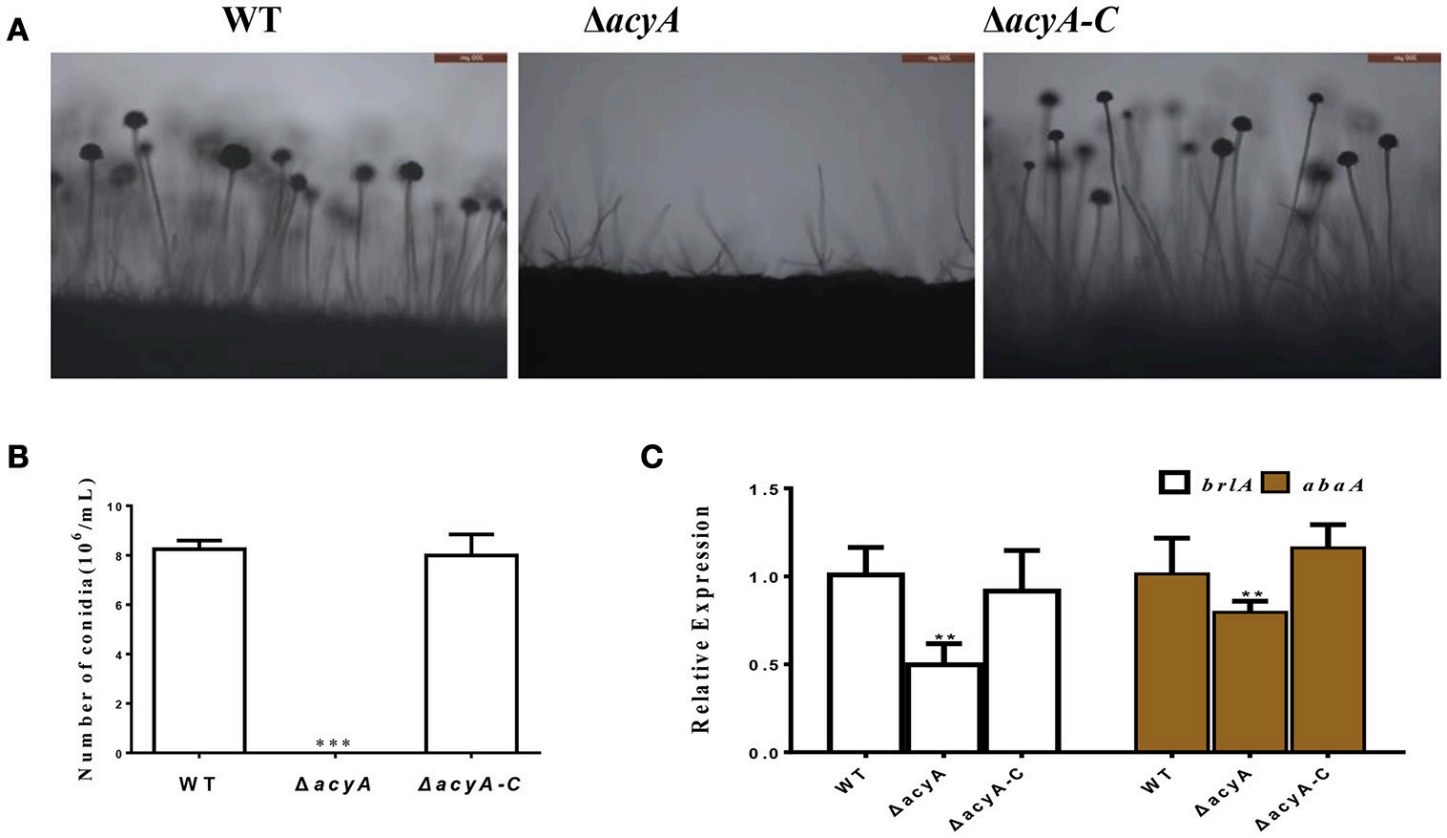
**Requirement of ***acyA*** for conidiation. (A)** Conidia formation was observed under a light microscope at 12 h after induction with illumination. **(B)** The number of conidia in wild-type and mutant strains were measured after grown on YES agar for 4 d at 37°C. **(C)** qRT-PCR revealed that the expression levels of conidia related gene *brlA* and *abaA* were decreased in the Δ*acyA* mutant. Values in **(B,C)** are the means plus standard errors (error bars) for three replicates. The asterisks ^**^ and ^***^above the bars represent significantly different (*p* ≤ 0.01 or *p* ≤ 0.001).

In addition to fungal growth, the Δ*acyA* mutant was found reduced severely in conidiation when compared with the wild-type or Δ*acyA-C* strain (Figure 4A). For further analysis of the defect in conidiation, we examined the conidiophore formation, and the result showed that the Δ*acyA* mutant failed to form normal conidiophore, and the wild-type phenotype could be basically recovered in the Δ*acyA-C* strain (Figure 4B). The transcript levels of the conidia-related genes *brlA* and *abaA* were also detected by qRT-PCR, and the result showed that the expression levels of these two genes were decreased significantly in the Δ*acyA* mutant compared to the wild-type and Δ*acyA-C* strain (Figure 4C). Taken together, these results reveal that AcyA is essential for hyphal growth and conidiation in *A. flavus*.

### AcyA is required for sclerotial reproduction in *A. flavus*

**Figure 5:**
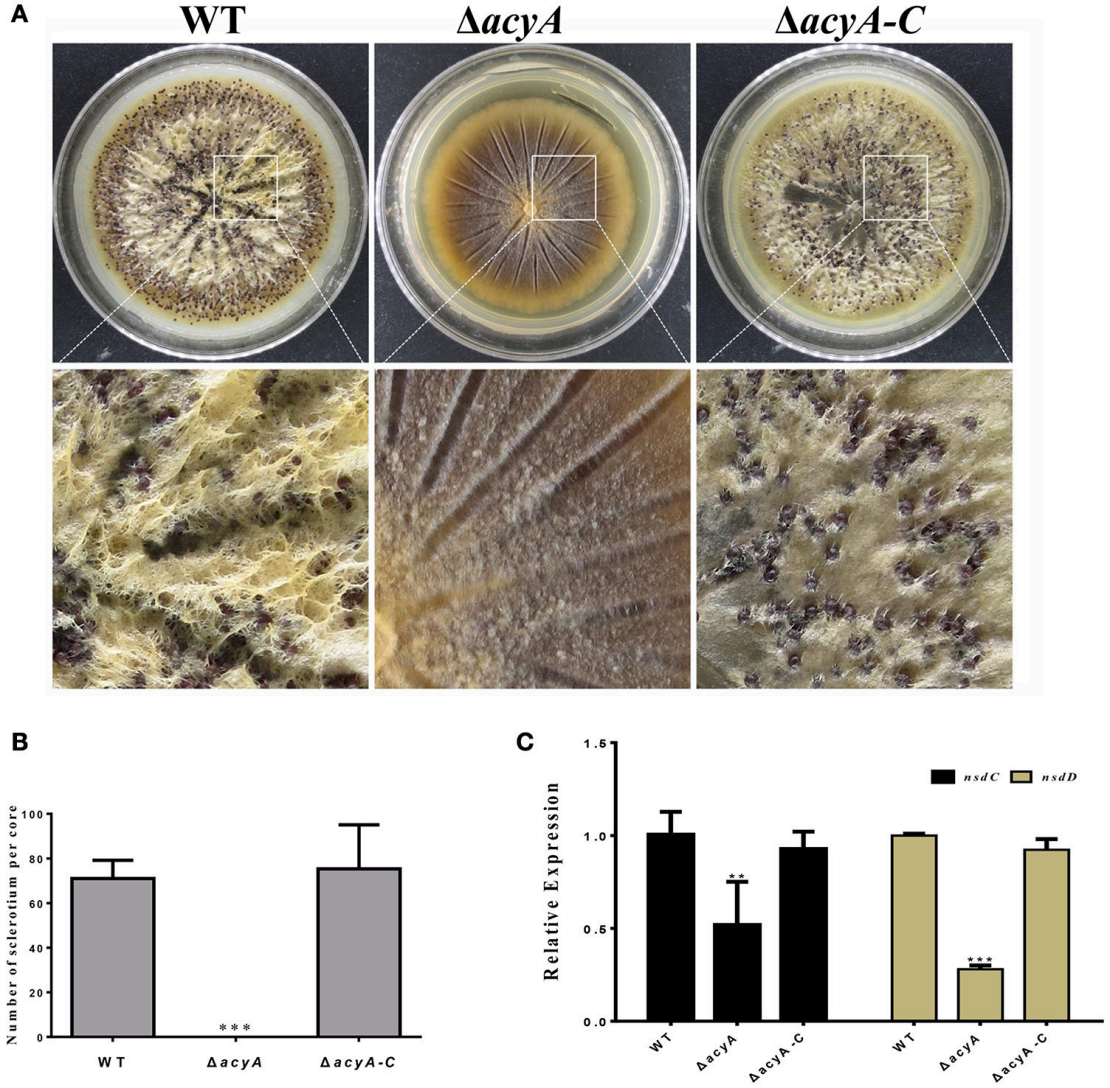
**Deletion of ***acyA*** blocked sclerotia reproduction in ***A. flavus***. (A)** Sclerotia production among the wild type, *acyA* deletion mutant and Δ*acyA-C* strain were observed after grown on WKM agar for 7 d in dark condition. The plates were sprayed with 70% ethanol to allow visualization of sclerotia. **(B)** Sclerotial production was counted from three replicates of WKM plates in **(A)**. **(C)** qRT-PCR revealed that the sclerotial related gene *nsdC* and *nsdD* were decreased in Δ*acyA* mutant. Values in **(B,C)** are the means plus standard errors (error bars) for three replicates. The asterisks ^**^ and ^***^above the bars represent significantly different (*p* ≤ 0.01 or *p* ≤ 0.001).

In order to adapt the stress condition, *A*. *flavus* is able to reproduce a survival structure sclerotia, which is considered to be a vestige of the cleistothecia. To determine if AcyA was involved in the formation of sclerotia, all the strains were grown on the sclerotia-inducing Wickerham medium at 37°C for 7 d. The results showed that sclerotia formation in Δ*acyA* mutant was severely blocked compared to that in wild type and Δ*acyA-C* strain (Figures 5A,B), indicating that AcyA is required for the formation of sclerotia in *A. flavus*. To further confirm this finding, we performed qRT-PCR to detect the transcript levels of the sclerotia-related genes, *nsdC* and *nsdD*, and the results showed that the expression levels of *nsdC* and *nsdD* were both significantly decreased in the Δ*acyA* mutant compared to that of WT and Δ*acyA-C* strains (Figure 5C), demonstrating that AcyA is necessary for the normal maturation of sclerotia.

### AcyA is involved in nutrient sensing

**Figure 6:**
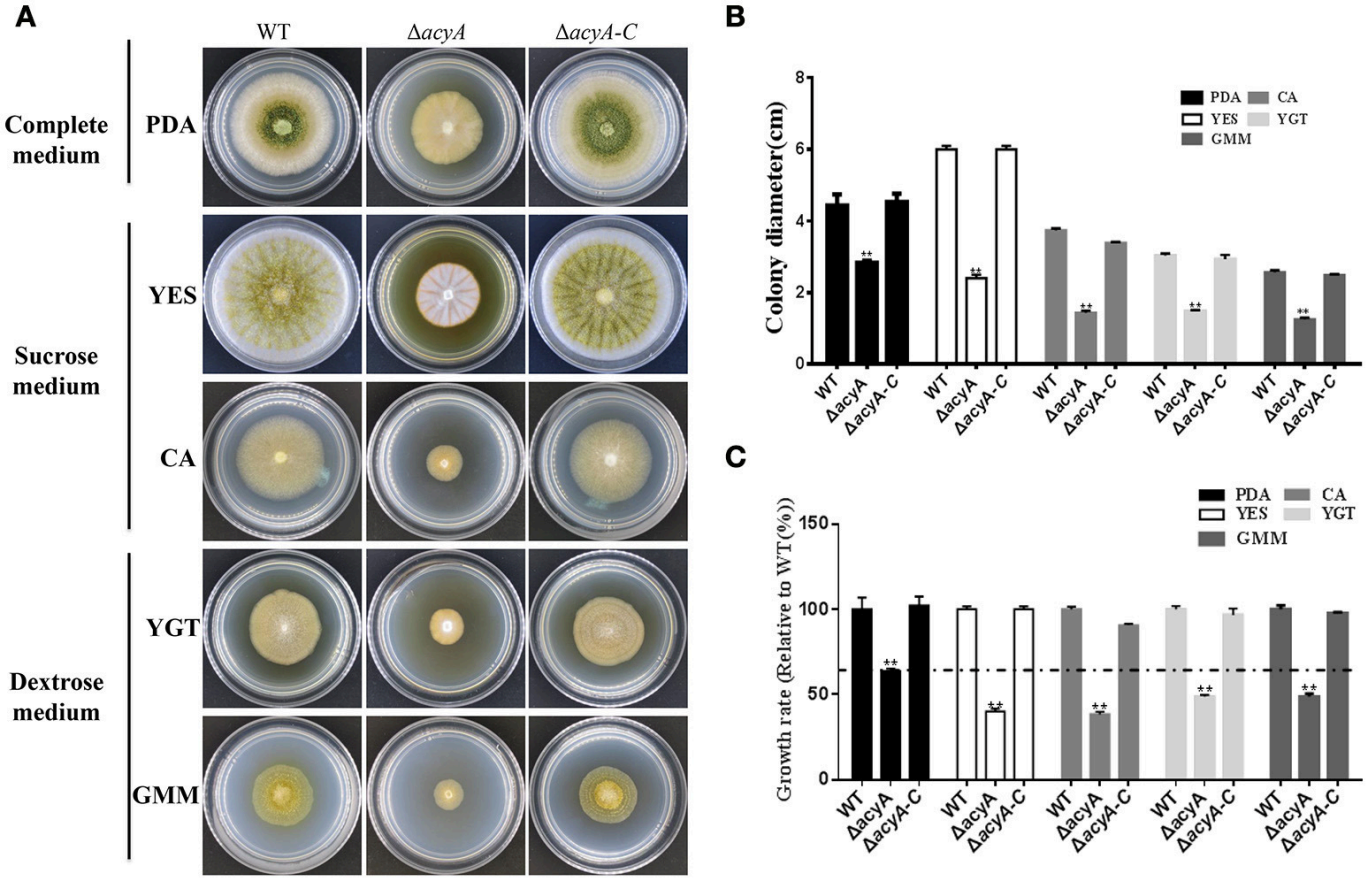
**Defects of the ***acyA*** mutant in phenotype and growth rate when grown on different medium. (A)** Phenotype of Wild-type (WT), Δ*acyA* and Δ*acyA-C* stains were grown on different medium for 4 d. **(B)** colony diameter of WT, Δ*acyA* and Δ*acyA-C* complemented strain on different cultures were assayed. **(C)** Growth rate of Δ*acyA* and Δ*acyA-C* strain relative to WT on PDA, YES, CA, YGT, and GMM media were measured, respectively. Values in **(B,C)** are the means plus standard errors (error bars) for three replicates. The asterisks ^**^above the bars represents significantly different (*p* ≤ 0.01).

In fungi, adenylyl cyclases are large proteins providing multiple points for signal sensing, including response to glucose and amino acids. To determine the function of *acyA* in sensing nutrient, we assay vegetative growth of *acyA* deletion mutant on different medium. As shown in Figure 6A, Δ*acyA* showed reduced aerial hyphae on all assayed media. When grown on nutrient-rich media PDA, Δ*acyA*, although defective in colony morphology, showed a largest colony diameter compared to other medium (*p* < 0.01; Figures 6B,C). When the cultures were replaced by the media which used the sucrose or dextrose as carbon source, Δ*acyA* was reduced 20~40% in colony diameter compare to the PDA media (Figure 6C), indicating that the *acyA* mutant was likely defective in nutrient sensing.

### Altered heat shock responses in the *acyA* mutant

**Figure 7:**
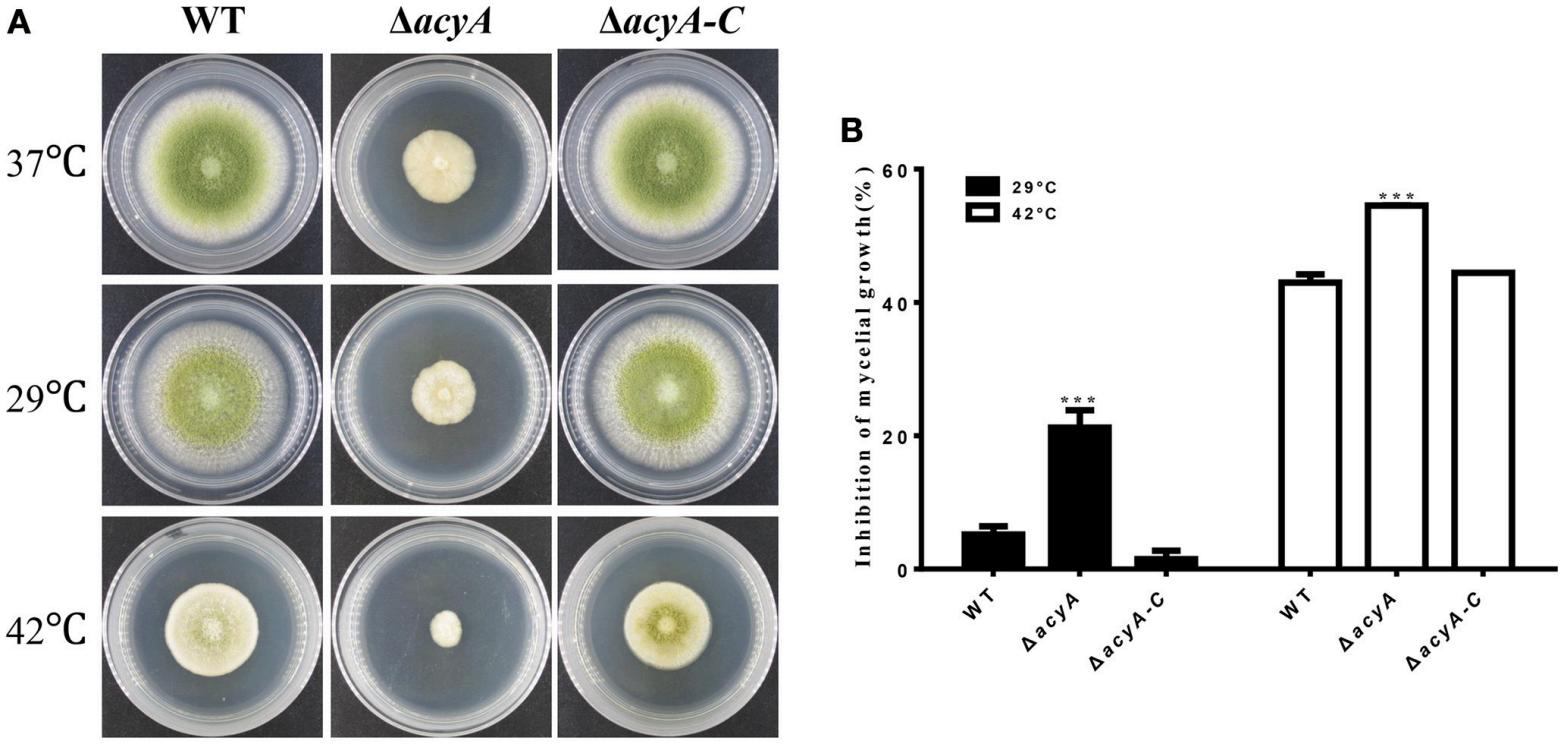
**The ***acyA*** mutant decreased tolerance to changing temperatures. (A)** Phenotype of Wild-type (WT), Δ*acyA* and *acyA*-*C* stains were grown on PDA medium at different temperature. Cultures were grown on PDA medium at 37°C for 2 d, then kept growing at 37°, 29°, or 42°C for 2 d. **(B)** Growth rate of hyphal growth at different temperature relative to that of 37°C were measured. Values in **(B)** are the means plus standard errors (error bars) for three replicates. The asterisks ^***^above the bars represent significantly different (*p* ≤ 0.001).

Because cAMP signaling is known to be involved in heat tolerance, we assayed hyphal growth at different temperature. As we can see in Figure 7, *A*. *flavus* hyphal grew fast at 37°C on PDA cultures (Figure 7A). When grown in the condition that is suitable for aflatoxin biosynthesis (29°C), the *acyA* mutant was reduced approximately 21.2% in growth rate, whereas the growth rate of WT and Δ*acyA-C* strains decreased slightly (Figure 7B). When the cultures were kept growing at 42°C for 2 d, the colony growth of Δ*acyA* was reduced almost 55%, while 42% for WT, and 44% for Δ*acyA-C* strain. These results indicated that the *acyA* mutant was much more sensitive to temperature stress than the wild-type for hyphal growth.

### AcyA is important for response to hyperosmotic stresses and oxidative stresses

**Figure 8:**
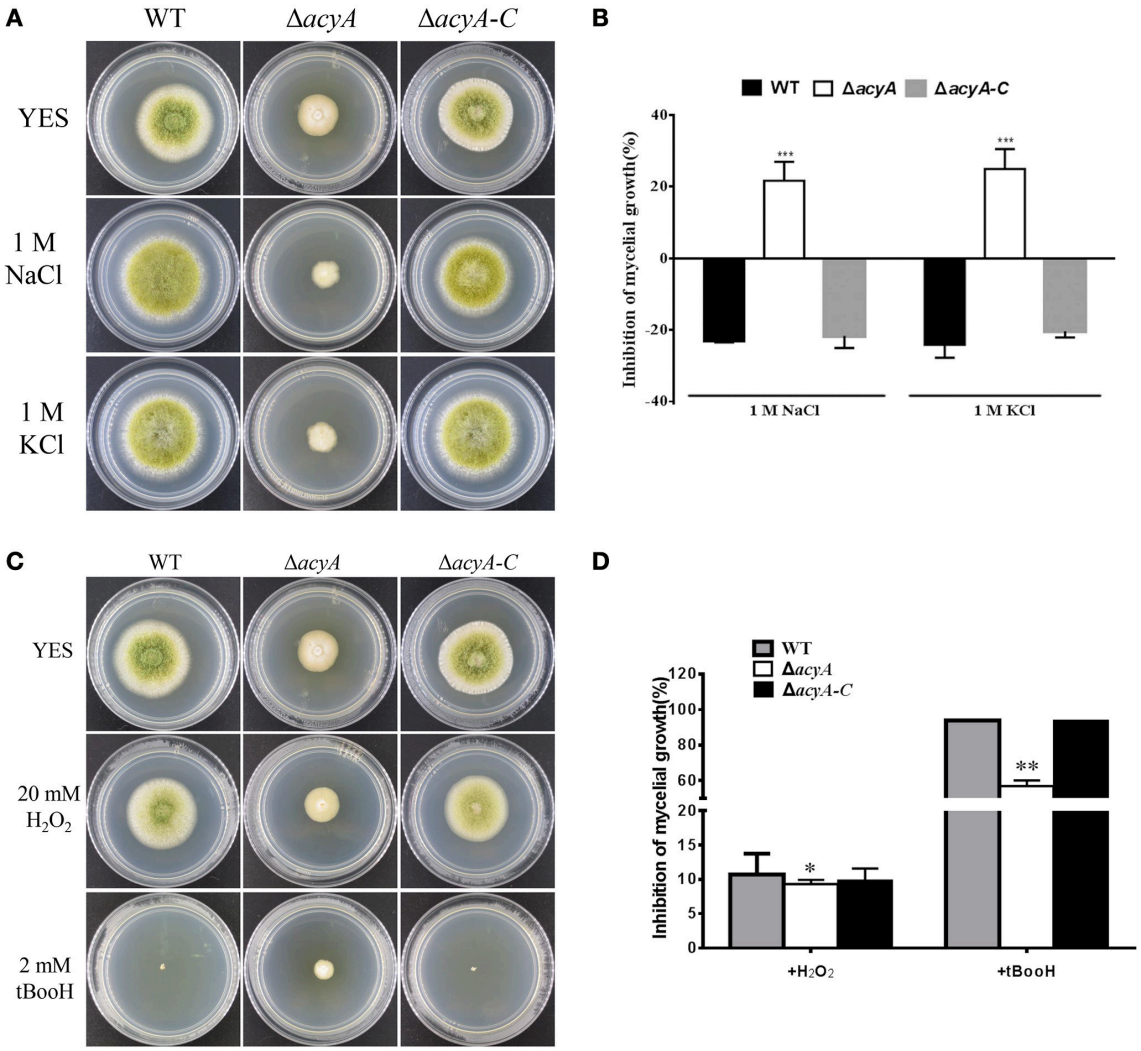
**Sensitivity of the WT, ***acyA*** mutant and Δ***acyA-C*** strain to hyperosmotic stresses and oxidative stresses. (A)** Phenotype of WT, Δ*acyA* and *acyA* complementation stains were grown on PDA medium supplemented with or without 1M NaCl and 1M KCl for 3 d. **(B)** The inhibition rate of the mycelial radial growth was quantified after incubation on media with 1M NaCl and 1M KCl for 3 d. **(C)** Colonies of WT, Δ*acyA* and *acyA* complementation stains were grown on PDA medium supplemented with or without 20 mmol/L hydrogen peroxide (H_2_O_2_) or 2 mmol/L tBooH (tert-butyl hydroperoxide) for 3 d. **(D)** The inhibition rate of the mycelial radial growth was quantified after incubation on media with H_2_O_2_ and tBooH for 3 d. Values in **(B,D)** are the means plus standard errors (error bars) for three replicates. The asterisks ^*^, ^**^, and ^***^above the bars represent significantly different (*p* ≤ 0.05, *p* ≤ 0.01, or *p* ≤ 0.001, respectively).

cAMP signaling plays a major role in regulating cellular responses to environmental signals. To test the ability of *acyA* in response to various environmental stress, we assayed the sensitivity of WT, Δ*acyA* and Δ*acyA-C* strains in response to osmotic stress and oxidative stress. As shown in Figures 8A,B, the growth of Δ*acyA* mutants was severely blocked after the addition of 1M NaCl or 1M KCl in PDA media compared to the WT and Δ*acyA-C* strain, indicating that AcyA is involved in responses to osmotic stresses in *A*. *flavus*. Since Δ*acyA* mutant exhibited increased sensitivity to osmotic stresses, we were also interested in determining the role of AcyA played in responses to other stresses. As we can see in Figures 8C,D, the wild-type and Δ*acyA-C* strain, but not Δ*acyA*, were much more sensitive to oxidative agent hydrogen peroxide. The growth of the wild-type and Δ*acyA-C* strains were severely blocked by the addition of tert-butyl hydroperoxide compared to the Δ*acyA* mutant, demonstrating that AcyA is likely to play a negative role in responses to oxidative stresses in *A*. *flavus*.

### AcyA is essential for aflatoxin biosynthesis

**Figure 9:**
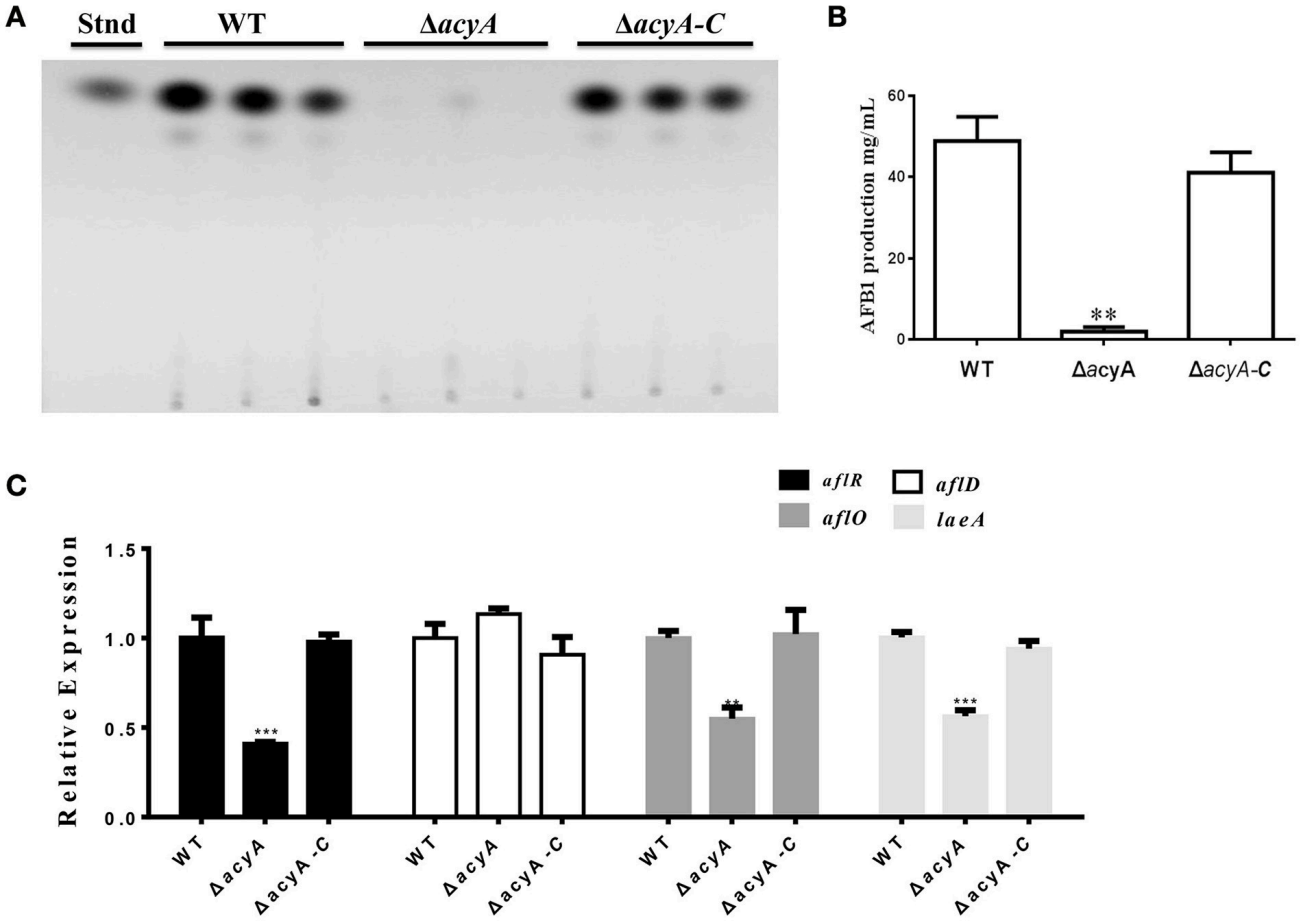
**Aflatoxin production of the wild type, ***acyA*** deletion mutant and Δ***acyA-C*** strain. (A)** Aflatoxin production was detected by TLC after cultured in GMM liquid media for 5 d at 29°C in the dark. **(B)** Quantification of AFB1 production as in **(A)**. **(C)** qRT-PCR results of the expression level of *laeA*, and the aflatoxin-related genes, *aflR, aflD*, and *aflO* from WT, Δ*acyA* and Δ*acyA-C* strains. Gene expression levels were normalized (ΔΔCT analysis) to *actin*. Line bars in each column denote standard errors of three experiments. The asterisks ^**^ and ^***^ represent significantly different (*p* ≤ 0.01 and *p* ≤ 0.01, respectively).

*Aspergillus flavus* is a well-known aflatoxins (AFs)-producing fungus, which are found to be one of the most toxic and carcinogenic natural contaminants. To examine the effect of AcyA on AF biosynthesis, AF production was tested from the WT, Δ*acyA* and Δ*acyA-C* strains by thin layer chromatography (TLC) at 5 d. The result indicated that the Δ*acyA* mutant failed to produce aflatoxin compared to WT and Δ*acyA-C* strains (Figures 9A,B). The qRT-PCR was also performed to detect the transcript levels of *laeA*, encoding a global regulator of many secondary metabolisms, the aflatoxin globally regulated gene *aflR*, and the structure genes, *aflD* (*nor-1*) and *aflO* (*omtB*), and the resluts showed that the expression levels of *laeA, aflR* and *aflO* were significantly decreased in the Δ*acyA* mutant compared to that in WT and Δ*acyA-C* (Figure 9C), however, the *aflD* transcript showed no difference among these strains (Figure 9C). All these results indicated that the AcyA might play an important role in regulating aflatoxins biosynthesis by reducing the AF regulator genes' expression in *A*. *flavus*.

### AcyA contributes to the full virulence to crop seeds

**Figure 10:**
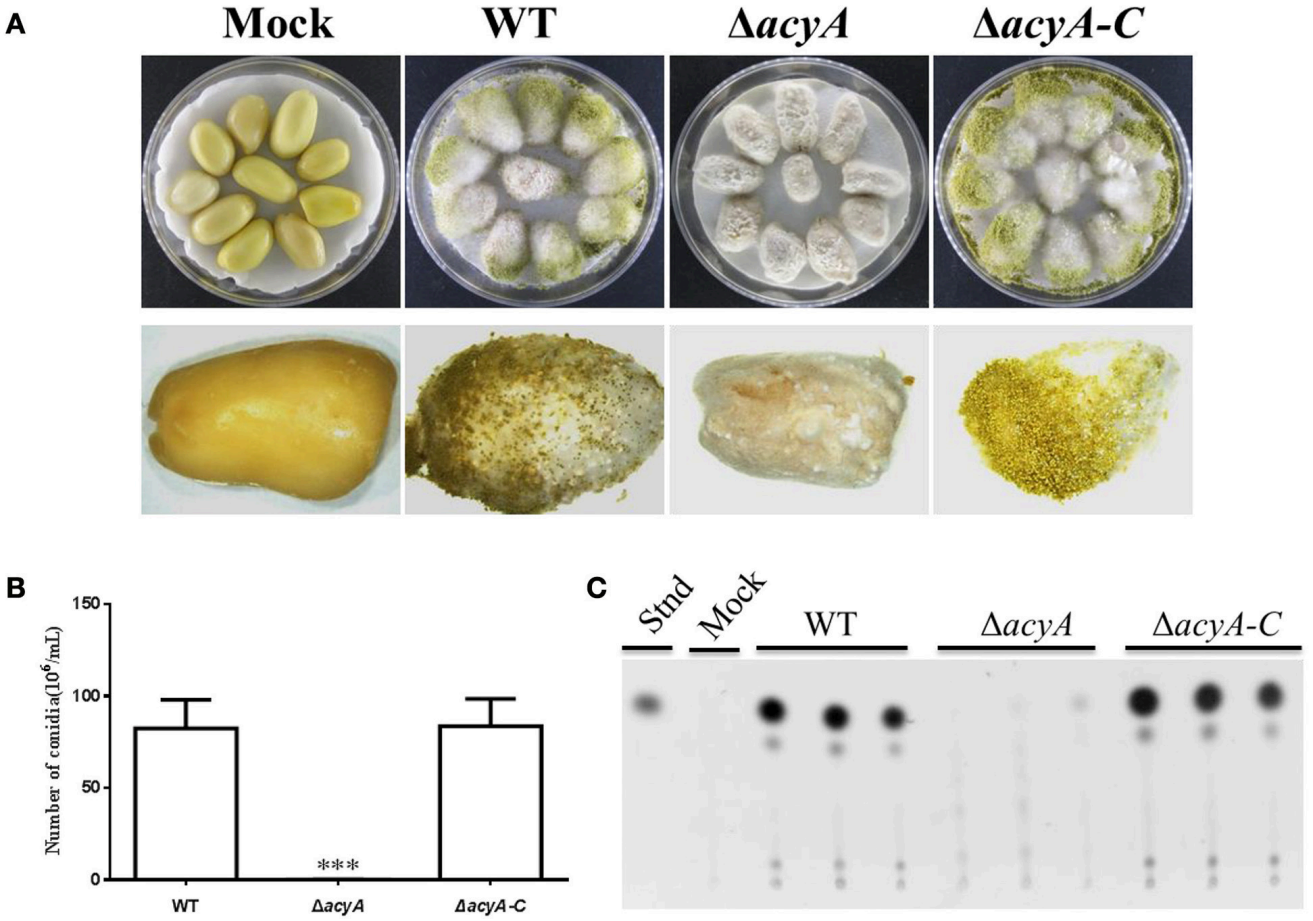
**Host colonization of the wild type, ***acyA*** deletion mutant and Δ***acyA-C*** strain. (A)** The strains were grown on peanut seed in the dark at 29°C for 7 d. **(B)** Conidia production was assessed from infected peanut seed. **(C)** Aflatoxin production were detected by TLC, which were extracted from infected peanut seed. Values in panel **B** are the means plus standard errors (error bars) for three replicates. The asterisks ^***^above the bars represent significantly different (*p* ≤ 0.001).

To determine the function of AcyA in pathogenicity, peanut seed was inoculated with spore suspension from the wild-type, Δ*acyA* and Δ*acyA-C* strains. The Δ*acyA* grew less vigorously than wild-type and Δ*acyA-C* strains on crop seeds (Figure 10A). Conidial production was also measured in these strains on seed, and the *acyA* deletion mutant was found fail to produced conidia compared to WT andΔ*acyA-C* strains (Figure 10B). We also detected the AF production from the infected peanut seed, which showed that the Δ*acyA* produced no detectable AF production compared to the WT and Δ*acyA-C* strain (Figure 10C). All these results indicated that AcyA contributed to the full virulence to crop seeds.

### AcyA synthesizes intracellular cAMP in *A. flauvs*

**Figure 11:**
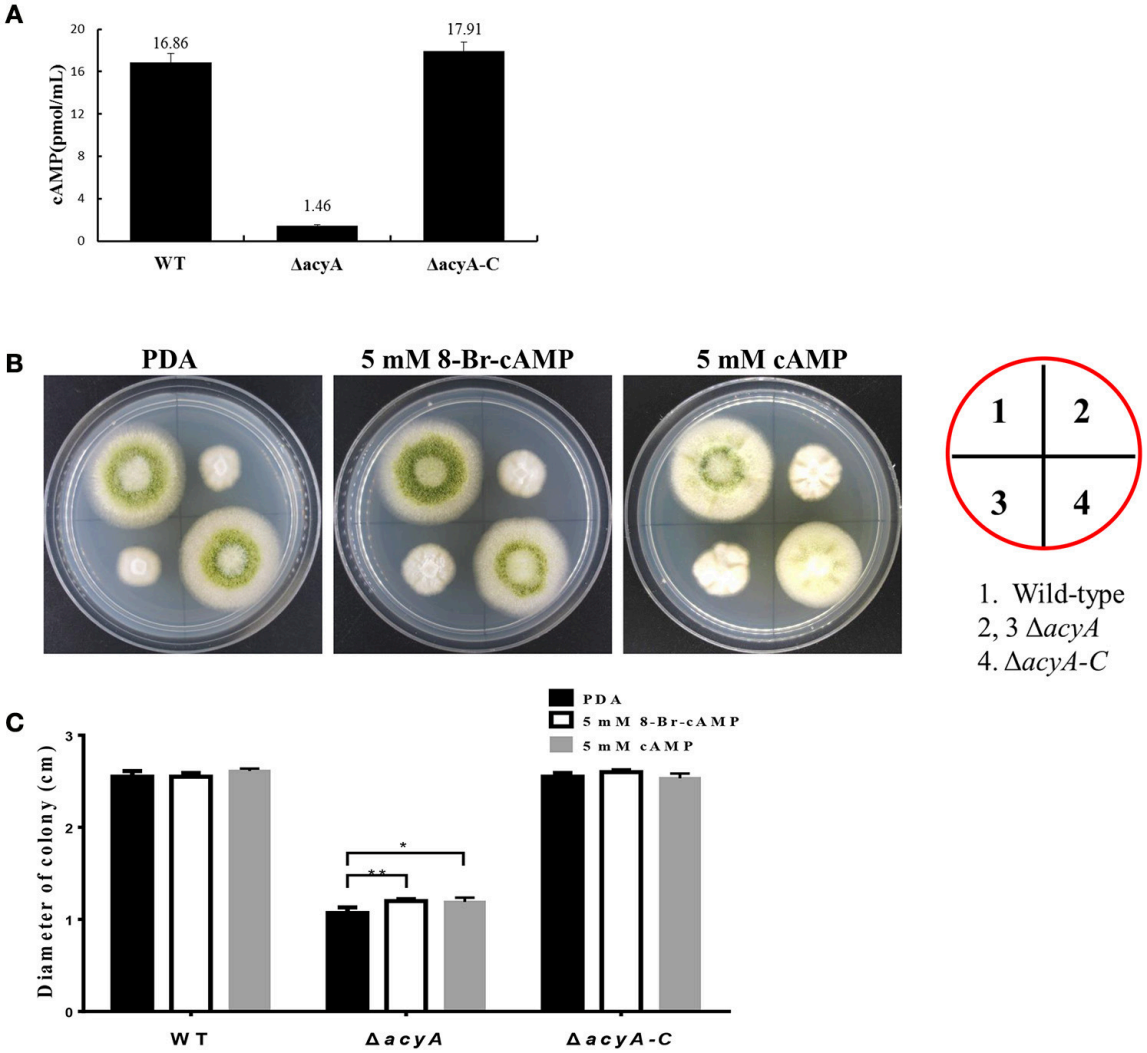
**AcyA is responsible for intracellular cAMP levels in ***A. flavus***. (A)** Inactivation of *acyA* leads to decreased accumulation of cAMP levels. **(B)** Hyphal growth of the wild type, *acyA* deletion mutant and Δ*acyA-C* strain on PDA media supplemented without or with 5 mmol/L cAMP or 5 mmol/L 8-Bromo-cAMP. **(C)** colony diameter of WT, Δ*acyA* and Δ*acyA-C* complemented strain on PDA media supplemented without or with 5 mmol/L cAMP or 5 mmol/L 8-Bromo-cAMP were assayed. Values in panels A and C are the means plus standard errors (error bars) for three replicates. The asterisks ^*^ and ^**^above the bars represent significantly different (*p* ≤ 0.05 or *p* ≤ 0.01, respectively).

In order to assess whether the phenotypic defects in *acyA* deletion mutant was due to decreased levels of cAMP, we quantified and compared the steady-state levels of cAMP from the wild-type, Δ*acyA* and Δ*acyA-C* strains. cAMP levels were measured after 2 d incubation in the dark, and the result showed that levels of intracellular cAMP in the Δ*acyA* mutant was reduced more than 10-fold compared to than that in WT and Δ*acyA-C* strains (Figure 11A). These results indicate that AcyA is responsible for synthesizing intracellular cAMP in *A. flavus*. Here, we also assayed whether exogenous cAMP or 8-Bromo-cAMP would recover the phenotype defects in Δ*acyA* mutant. As shown in Figures 11B,C, the hyphal growth of the Δ*acyA* mutant showed a significant increase in the presence of 5 mM cAMP or 8-Bromo-cAMP, but showing no difference in the wild-type or Δ*acyA-C* strains. However, neither the exogenous cAMP nor 8-Bromo-cAMP could fully recover the defects in Δ*acyA* mutant, indicating additional roles for AcyA, which is a multi-domain protein, each of which might be important for its full function, not just synthesizing cAMP in *A*. *flavus*.

## Discussion

cAMP signaling pathway has been shown to be very important for nutrient sensing, asexual development and secondary metabolism in fungi (Shimizu et al., 2003; Grosse et al., 2008; Zhang et al., 2011; Hu et al., 2014). In this study, we functionally characterized *acyA* gene in *A. flavus*, and found that the acyA deletion mutant wasseverely reduced in aerial hyphal and radial growth. In *A*. *nidulans* and *F. verticillioides*, a reduction in growth of the adenylate cyclase gene deletion mutants was found, which was similar to that of Δ*acyA* in *A. flavus*(Fillinger et al., 2002; Choi and Xu, 2010). In *N*. *crassa*, inactivation of *cr-1* was also severely blocked in vegetative development (Terenzi et al., 1979). However, in *M*. *oryzae*, the aerial hyphal of *MAC1* deficient mutant was found significantly reduced, but showing no difference in radial growth (Choi and Dean, 1997; Adachi and Hamer, 1998). Therefore, although the role of adenylate cyclase is conserved and important in fungal growth, but remains different in various fungi.

In *Aspergillus*, The cAMP/PKA pathway has been shown to have a major role in stimulating vegetative growth and repressing conidiation (Shimizu and Keller, 2001; Krijgsheld et al., 2013). However, our study, suggests that AcyA is required for conidiation and sclerotia formation. Here, we found that inactivation of *acyA* in *A*. *flavus* failed to produce conidiation, and concurrently decreasing in expression of two conidia-specific genes *brlA* and *abaA*. A defect in sporogenous structure was also found in the Δ*acyA* mutant, which might result in abnormal sporulation in the mutant. Although the negative role of cAMP/PKA pathway acts in asexual development in *Aspergillus*, defect in sporulation is a common event found in the adenylate cyclase deficient mutants of previously known filamentous fungi. As we know, inactivation of AcyA, leading to a low level of intracellular cAMP, causes a severe defect in fungal growth, which might also affect conidia formation concurrently, which has to gone through a period of vegetative growth that is necessary for cells to acquire the ability to respond to development signals (Lee et al., 2016). Former studies had also shown that conidial and sclerotial production were likely to remain balanced (Amaike and Keller, 2011). Here, we found that inactivation of *acyA* in *A*. *flavus* failed to form sclerotia. We also found that deleting the *acyA* gene led to lower transcript levels of sclerotia formation related genes *nsdC* and *nsdD*, which might lead to abnormal sclerotia formation. In *S. rolfsii* and *R. solani*, exogenous cAMP were shown to alter their sclerotial production (Rollins and Dickman, 1998). In *R. solani*, cAMP has been shown to enhance sclerotial development even in non-sclerotium-forming strains (Rollins and Dickman, 1998). Aberrant sclerotia were also found in adenylate cyclase *sac1* gene deletion mutant in *S*. *sclerotiorum* (Jurick and Rollins, 2007). These data indicate that cAMP signaling is likely to play an important role in regulating conidiation and sclerotial formation.

Former studies have indicated that cAMP/PKA pathway is likely to negatively regulate AF biosynthesis in *A*. *flavus*. However, the roles of adenylate cyclase in AF or other secondary metabolism are yet characterized in *Aspergillus*. In this study, we found that inactivation of *acyA* results in a severe reduction in intracellular cAMP levels and concurrent decrease in AF production. It is interesting to wonder why a block of cAMP signaling by deleting *acyA* from *A*. *flavus* causes reduction in AF biosynthesis and its related genes' expression. There are two main reasons for the decreasing AF production in *acyA* deficient mutant. One for this, we think, is that inactivation of *acyA* led to severe defects in *A*. *flavus* development, which might affect AF biosynthesis. In the other hand, we hold the idea that deletion of *acyA* might alter the activated state of cAMP dependent PKA in the cAMP signaling pathway, which might affect the transcriptional level of *aflR*. In *A*. *nidulans*, activating the G-protein α-subunit, FadA, or PkaA (PKA catalytic subunit) inhibits *aflR* expression and subsequent *stc* expression. Previous studies also showed that exogenous cAMP and IBMX promoted AF production in *A*. *parasiticus*, while the treated cultures were found decreased in PKA activities (Roze et al., 2004a,b). Therefore, we hypothesis that, a block in AF production in the Δ*acyA* mutant might be caused by the changing activities of PKA, leading to the changing expression levels of AF related genes in *A*. *flavus*.

In many phytopathogenic and animal pathogenic fungi, like *M. oryzae, C. neoformans* and *F. verticillioides*, the cAMP-PKA pathway is known to function in virulence (Alspaugh et al., 2002; Choi and Xu, 2010). To address the effect of AcyA on *A*. *flavus* pathogenicity, we detected host colonization in the Δ*acyA* mutant. The Δ*acyA* mutant shows significantly reduction in pathogenicity, which might be caused by a number of factors. Firstly, deletion of *acyA* results in a defect of vegetative growth and conidiation, leading to the mutant grow less vigorously on the crop seeds compared to the wild-type. In *A*. *flavus*, the virulence is thought to be multifactorial and is mainly connected with development, sporulation, secondary metabolism, and other conditions(Amaike and Keller, 2011). In this study, we found that the the Δ*acyA* mutant fail to produce the secondary metabolism, aflatoxins, which would impair the pathogenicity of *A*. *flavus* on crop seeds. The roles of adenylate cyclase seem to be conserved in many other plant pathogenic fungi, just like *M. oryzae, S*. *sclerotiorum* and *F. verticillioides*, for which the adenylate cyclase gene is required for their pathogenesis. Given these properties, targeting cAMP/PKA pathway could be a good strategy to control *A*. *flavus* contaminating preharvest and postharvest seed crops.

## Conclusions

In summary, our results provide evidence that adenylate cyclase AcyA is responsible for cAMP synthesis in *A. flavus*, and showing that AcyA has pleiotropic effects on growth, conidiation, virulence and AF biosynthesis. Our results also provide valuable information that could advance our understanding of the cAMP signaling in AF biosynthesis and would provide strategies to block AF production or *A*. *flavus* invasion in preharvest and postharvest agriculture crops.

## Author contributions

KY, QQ, and SW designed the experiments and wrote the manuscript. KY, QQ, YL, and LL performed all the experiments. CC and FZ performed a few experiments and data analysis. All authors read and approved the final manuscript.

### Conflict of interest statement

The authors declare that the research was conducted in the absence of any commercial or financial relationships that could be construed as a potential conflict of interest.
