# Supplementary material for: Adenylate Cyclase AcyA Regulates Development, Aflatoxin Biosynthesis and Fungal Virulence in Aspergillus flavus
Source: Front Cell Infect Microbiol. 2016 Dec 21;6:190. doi: 10.3389/fcimb.2016.00190 (PMC5175447; doi:10.3389/fcimb.2016.00190)
Supplement: Supplementary file 1 [file Presentation1.PDF]

The adenylate cyclase AcyA regulates development and aflatoxin biosynthesis in *Aspergillus flavus*

Kunlong Yang[§], Qiuping Qin[§], Yinghang Liu, Limei Zhang, Linlin Liang, Huahui Lan, Chihao Chen, Zhenhong Zhuang, Feng Zhang, Shihua Wang*

Key Laboratory of Pathogenic Fungi and Mycotoxins of Fujian Province, Key Laboratory of Biopesticide and Chemical Biology of Education Ministry, and School of Life Sciences, Fujian Agriculture and Forestry University, Fuzhou 350002, China

§These authors contributed to this work equally.

* Author correspondence to Shihua Wang. E-mail: wshyyl@sina.com; Tel and Fax: +0086 (591) 87984471

```
AcyA_A.fumigatus_      1 ------------------------------------------------------------
AcyA_A.clavatus_       1 ------------------------------------------------------------
AcyA_A.niger_          1 ------------------------------------------------------------
AcyA_A.flavus_         1 ------------------------------------------------------------
AcyA_A.oryzae_         1 ------------------------------------------------------------
AcyA_A.nidulans_       1 ------------------------------------------------------------
AcyA_N.crassa_         1 ------------------------------------------------------MTRNDG
AcyA_C.albicans_       1 ------------------------------------------------------------
AcyA_S.cerevisiae_     1 ------------------------------------------------------------
consensus              1


AcyA_A.fumigatus_      1 ------------------------------------------MSLRGSENSHRRRSSE--
AcyA_A.clavatus_       1 ------------------------------------------MSMRDSDQSLGRRSSE--
AcyA_A.niger_          1 ------------------------------------------------------------
AcyA_A.flavus_         1 ------------------------------------------MSFQGGDIPEGRRSSE--
AcyA_A.oryzae_         1 ------------------------------------------MSFQGGDIPEGRRSSE--
AcyA_A.nidulans_       1 ------------------------------------------MAFPGGELPEGRHSSE--
AcyA_N.crassa_         7 GSRYSSIDSAQSSITAKPFPLTPTSSLGSSMTRPLSPSLQAGSSSSNGGHNVSRSASQGA
AcyA_C.albicans_       1 ------------------------------------------------------------
AcyA_S.cerevisiae_     1 ---------------------------------------MSSKPDTGSEISGPQRQEE--
consensus             61                                           ..  . .    .....


AcyA_A.fumigatus_     17 --------------------SSGAHSLLSQETVREIQNPRPLYNTVMPNGRSNSPFPRSA
AcyA_A.clavatus_      17 --------------------SSGKNSWLSQETVREGPSQQPLDRQAMPNGRSNSPFPGPV
AcyA_A.niger_          1 ------------------------------------------------------------
AcyA_A.flavus_        17 --------------------SSGGNSWLSQETVKDNQTNRYQRNHAMLNGRLISPFSGFA
AcyA_A.oryzae_        17 --------------------SSGGNSWLSQETVKDNQTNRYQRNHAMLNGRLISPFSGFA
AcyA_A.nidulans_      17 --------------------SSGNNSGLSQETIRDDGRR---YGRIVPSGRSISPHSKDD
AcyA_N.crassa_        67 RRPAPTRLKTDDGTQFASRSSRDFESSQSQSQPSHTQTRSHSLSQATLQSCSQPQLSLNS
AcyA_C.albicans_       1 ------------------------------------------------------------
AcyA_S.cerevisiae_    20 ------------------QEQQIEQSSPTEANDRSIHDEVPKVKKRHEQNSGHKSRRNSA
consensus            121                     ... .. ........          . ...  ..


AcyA_A.fumigatus_     57 GLERSASQGH--------NQPRQGSSNNLYSSTFSWNSPDISPTDQRR--GVG--RKAMA
AcyA_A.clavatus_      57 NMERSASQGH--------RPPNMGSANNLYASNFSWGSPDISPTDQRK--GLGNHRKVMA
AcyA_A.niger_          1 ----------------------------------------------------------MN
AcyA_A.flavus_        57 KADRSTSQGR--------KQPPTLGAHDLSTSNFTWPSPDVSPTDKRKDSGFTGHRKSSA
AcyA_A.oryzae_        57 KADRSTSQGR--------KQPPTLGAHDLSTSNFTWPSPDVSPTDKRKDSGFTGHRKSSA
AcyA_A.nidulans_      54 GSDRLELPAP--------RPPSLGAPNDLYTSMFSWGSPDISPTDVRKNQGFGSHRKALA
AcyA_N.crassa_       127 QQSQSPSQYHSQQTPTQQQPQQQVSPTGGSRLTQSPTTPSNASIREHRMSELGGYRREMA
AcyA_C.albicans_       1 ----------------------------------------------------------MS
AcyA_S.cerevisiae_    62 YSYYSPRSLSMTKSRESITPNGMDDVSISNVEHPRPTEPKIKRGPYLLKKTLSSLSMTSA
consensus            181   ... ....          .       .  . ... ........ ..  .    .. ..


AcyA_A.fumigatus_    105 TSGADDPGPPP-TSSSSSKQLQLN-PNQIDYFHQNPNASWSSS----------PKIAPMD
AcyA_A.clavatus_     107 LLGNDDPGPPP-TSSSNTRSSQPNQPNQIDYFHQDPNASWSSS----------PQIAPMD
AcyA_A.niger_          3 LLGTEDPGTSP-TANSNNRPPQLN-LNPPDNPQHDAEPSLASS----------PTVAPND
AcyA_A.flavus_       109 AIGPDEPGPPP-TFSSSPRISCPN-SDEVDYFQQDPNASYSSS----------PKIAPGD
AcyA_A.oryzae_       109 AIGPDEPGPPP-TFSSSPRISCPN-SDEVDYFQQDPNASYSSS----------PKIAPGD
AcyA_A.nidulans_     106 VLGNDDPGPPP-INNPSPR---VN-TNYGDYFQHDPQGTLSYS----------PRLGTGD
AcyA_N.crassa_       187 AMLDTTGGALSRTSSHSQQQPQQPQQQQQQQQQQQGHAAGSVSGTFSNLSQYAPYLGGNN
AcyA_C.albicans_       3 FLRRDKSK----------------------------------------------------
AcyA_S.cerevisiae_   122 NSTHDDNKDHGYALNSSKTHNYTSTHNHHDGHHDHHHVQFFPNR--------KPSLAETL
consensus            241  .. ....... . ... .    .  .  ..............          . ... .


AcyA_A.fumigatus_    153 SS----GTFSHDFSE--QEASPASATFRPSTGRTFASEP-----VDFDYNRDDRRPSVAS
AcyA_A.clavatus_     156 AP----GTFFHDLSE--HEASPASGSFRPGTGRTFASEP-----VDLDYNGDHRRPSIAS
```

```
AcyA_A.niger_          51 GP----STFFNDYSE--QEASPASNLFRPGTAQTYASES-----LELDYNGDHRRPSVAS
AcyA_A.flavus_        157 GN----GAFFQDHSE--HEASPASTSFRPGTSRTYASEP-----LELDYNGDHRRPSVAS
AcyA_A.oryzae_        157 GN----GAFFQDHSE--HEASPASTSFRPGTSRTYASEP-----LELDYNGDHRRPSVAS
AcyA_A.nidulans_      151 AH----GTFFHDHSE--HEASPATATFRPGTGRTLASDA-----PDLDYNGDHRRPSVAS
AcyA_N.crassa_        247 GGGMSMGSFLNDSTDNLSVISQLSPGIRPMTARPSQASAGSMEFPDSAYYDDERRPSIAS
AcyA_C.albicans_       11 ------ANFRDGSATGLEEPVSPTTHFSPNVPPP----------LDGNHGDHYHDPDSPR
AcyA_S.cerevisiae_    174 FKRFS-GSNSHDGNKSGKESKVANLSLSTVNPAPANRKPSKDSTLSNHLADNVPSTLRRK
consensus             301 .     .... . ..  ....... ..............     ................


AcyA_A.fumigatus_     202 ATTISS-QGSKSSTGGRFRKKLQGFFGDEYLPPGESKQEQEQDTRSLSSRPASIDHFKAR
AcyA_A.clavatus_      205 ATTISS-QGSKSSTGGRFRKKLQGFFGDEYLTPADSKQDSEQGAPSLNSRAGSIDQFRAR
AcyA_A.niger_         100 AMTVSS-QGSKSSAGALFKKKLQGFFGDEYVGNGDPRHDSDNFTQNSATKPSSLDQFRAR
AcyA_A.flavus_        206 ATTISS-QGSKSSTGGRFRKKLKGFFGEDYLP-GDSKLESDNGSQHPTKKSSLSEQHIFR
AcyA_A.oryzae_        206 ATTISS-QGSKSSTGGRFRKKLKGFFGEDYLP-GDSKLESDNGSQHPTKKSSLSEQHIFR
AcyA_A.nidulans_      200 ATTVSS-QGSKSSTSGLFRKKLQGFFGDDPNA-SDSKQDHDG-HHSSGSKPSSIDYFRSR
AcyA_N.crassa_        307 ITTTASSQGSRTSKTRGGLQKLQQFFGDRDDWPGRDSSEISLPQPSHSGPMSTGKEHRSH
AcyA_C.albicans_       55 SSVVSLPQLIHNSATHHLKENYRGFHAN-------------KRPKGIANVPPLAQPIKPR
AcyA_S.cerevisiae_    233 VSSLVRGSSVHDINNGIADKQIRPKAVAQLENTLHSSDVPNSKRSHRKSFLLGSTSSSSS
consensus             361 ...... ......................  .... . ..      .. .. .  . .


AcyA_A.fumigatus_     261 ERANSDGARNFPERSQDDTSSLHPS--------------------RPRTPLPSSEITPWL
AcyA_A.clavatus_      264 ERANSDGARNFPERSNDDASSRHPS--------------------RPRTPLPSSEITPWL
AcyA_A.niger_         159 ERANSDGSRNP-----GTSPAPNQA--------------------RPHTPLPSSEITPWL
AcyA_A.flavus_        264 ERANSDGARSPPQRLIGESSPQRP---------------------RPRAPLPSSEITPWV
AcyA_A.oryzae_        264 ERANSDGARSPPQRLIGESSPQRP---------------------RPRAPLPSSEITPWV
AcyA_A.nidulans_      257 QRADSEGSRRPSDGVQDDAH--QPG--------------------RPRTPLPSSDITPWE
AcyA_N.crassa_        367 SYSLPGSGRSHRDRNYSNATDHHPSTFGSVSTVGGRDRDASPVPSRPRTPVPAPEVVPFL
AcyA_C.albicans_      102 FKKKSNS-------------------------------------------LLNKLIYST
AcyA_S.cerevisiae_    293 RRGSNVSSMTNSDSASMATSGSHVLQHN------------------VSNVSPTTKSKDSV
consensus             421 .........   ..   ...  ..                     ...............


AcyA_A.fumigatus_     301 YQSFNDIPQFGEAPIREVPIAPEGSRLAGQTATNSGAPREARRHHGSHRHSRSKEEKPTV
AcyA_A.clavatus_      304 YQSFNDIPQFGEAPVREAPIASGSHRAAGQSATNAGASRDARRHFTGHRHSRSKEEKPTV
AcyA_A.niger_         194 YQSFNDIPQYGEAPVREAPTTTADGQRGSVSQRDQ-----SRRHFSGHRHSRSKEEKPTA
AcyA_A.flavus_        303 FQSFNDIPQYGEAPVREAPIGADGRRAAAQMARGS-QRNQTGRQFSGHRHSRSKEEKSTT
AcyA_A.oryzae_        303 FQSFNDIPQYGEAPVREAPIGADGRRAAAQMARGS-QRNQTGRQFSGHRHSRSKEEKSTT
AcyA_A.nidulans_      295 YQRYNDIPQLGEAPVREAPTALDRHRIGSQGASST-SRDPTRRHFSGHRHSRSKEEKANA
AcyA_N.crassa_        427 YQEADDIARYGEAPVRTSLTGPDRDRYIDSSQNPP---KTSSSARSGHSIVHLPGHHKHN
AcyA_C.albicans_      118 KKEDDEAATSGKESRSSSIISDEKRKSASSASSGS---------------SRQKFRFSSF
AcyA_S.cerevisiae_    335 NSESADHTNNKSEKVTPEYNENIPENSNSDNKREATTPTIETPISCKPSLFRLDTNLEDV
consensus             481 .................... . .. . . .  .    . . ............ .


AcyA_A.fumigatus_     361 AGDLAGYPARPATGRDDSYLGLRPFRDHSLNASTAMSSTTTLGGRSTSPTPSIQSAYSRE
AcyA_A.clavatus_      364 AGDLAGYPARPATGRDDSYLGLRPFRENSLNMPTAMSSSTTLGGRSTSPTPSMQSAYSRE
AcyA_A.niger_         249 AGDLAGYSNRPATGRDDLSLGLRPSREGSLNYPTPMTSTTTLVGRSTSPTPSVQSAYSRE
AcyA_A.flavus_        362 ADDITVYPSRPTTGRDDFGIGLR---ANSLNN-SAMNSTSTLV-RSTSPTPSMQSAHSRE
AcyA_A.oryzae_        362 ADDITVYPSRPTTGRDDFGIGLR---ANSLNN-SAMNSTSTLV-RSTSPTPSMQSAHSRE
AcyA_A.nidulans_      354 AGDLAGYPDRPSTGRDDFSVGLRPSRDGSLGFRPAANSSINLAGRSTSPTPSLQSFYTKD
AcyA_N.crassa_        484 KSNEDPRALKPSLSREDSAASFARDFRNGSSSMMGTRSRAQSPAPSWTGTSRGLKANSIS
AcyA_C.albicans_      163 DSNLSTSSSSPPKDK-----------KVSVSDTVSDSSTVTASMSNMPTISIDLNLDEMH
AcyA_S.cerevisiae_    395 TDIKTKTVPPTAVNSTLNSTHGTETASPKTVIMPEGPRKSVSMADLSVAAAAPNGEFTSTS
consensus             541 . ..  .. .. .....  ....    ...  ... ..... ............. ...


AcyA_A.fumigatus_     421 QSQNSPGAPS-NKRSILDKIRRTKAHGPLKHFPGSKSAQEASKSSS-KLARREASP-RRG
AcyA_A.clavatus_      424 QGQNSPGAPP-NKRSILDKIRRTKAHGPLKHFPGSKIAQEASRSSS-KLSRREASP-RRG
AcyA_A.niger_         309 QGQNSPGAQT-SKRSFLDKIRRPK-HGHLKNITGSKAVQETTKAAASKLSRREASPARRG
AcyA_A.flavus_        417 QGQSSPGTQLPNKRSILDKIRRPKAHGPLKHFPGAKGVQEA-KSTS-KLARRDVSPARRG
AcyA_A.oryzae_        417 QGQSSPGTQLPNKRSILDKIRRPKAHGPLKHFPGAKGVQEA-KSTS-KLARRDVSPARRG
```

```
AcyA_A.nidulans_      414 SGQGSPGAPS-SKRSFLGKLRRPN----LKHFPGSKGPTDAIRGTS-KLARRDASPGRRG
AcyA_N.crassa_        544 DGTSSP-APS-HKKGILGRFRRHN----KDKEDGSSLRSGSNHTLVHRPSRQDLTRAAES
AcyA_C.albicans_      212 DIIKSPETPT--------------------------------------------------
AcyA_S.cerevisiae_    455 NDRSQWVAPQ--SWDVETKRKKTKPKGRSKSRRSSIDADELDPMSPGPPSKKDSRHHHDR
consensus             601 ... ......  .......... . .. ........  ... .... .. ... ......


AcyA_A.fumigatus_     478 RQGS--LEGGTSLRNLDLGDYERRKDGKGIVMGSTKLRNRRGLGNEAPPG----------
AcyA_A.clavatus_      481 RQGS--LEGGASIRNLEIGDPERRKDGKGLAIGSAKLRNRRAVVNEAPPG----------
AcyA_A.niger_         367 RQGS--IESGTSSRLADSGDNERKRDGKGLAISSAKLRGRRGHANEVPPG----------
AcyA_A.flavus_        475 RQGS--LEGTTTPKTGD--ESDRKKDGRGLAISSAKLRGRRVLGTDTPSK----------
AcyA_A.oryzae_        475 RQGS--LEGTTTPKTGD--ESDRKKDGRGLAISSAKLRGRRVLGTDTPSK----------
AcyA_A.nidulans_      468 RQGS--LEG-APSKGAENGEHERKKDGKGLGIATGKLRGRRGAGHETPIG----------
AcyA_N.crassa_        598 TYPASVYVSDPSEQREVPVRPGYVRQTTAPGFTTKLFTSKKSSSAKQPQDD---------
AcyA_C.albicans_      222 ---------------PTAGLPTQKAEKKASPTAIKNWQAPESWDVKAPIK----------
AcyA_S.cerevisiae_    513 KDNESMVTAGDSNSSFVDICKENVPNDSKTALDTKLVNRLKSNLAMSPPSIRYAPSNLDG
consensus             661 ....   ...   .  .   .  .. ..........  ........    .  . *

                            Adenylate cyclase G-alpha binding
AcyA_A.fumigatus_     526 ---KDVKLSEEPG-----VWRLDTDLSHMEGIVSQQPPPSPGDKSKAQDGVIPKHEEGKK
AcyA_A.clavatus_      529 ---RDARPSDEQG-----VWRLDTDLSHMEGIVTQHPPPSPTDMGRPPDGIAVQQEDGKR
AcyA_A.niger_         415 ---KEIRQPDRSN-----VWELDTDLTHMEGIVN---PPSPSAKSKTFDGASIRSEETRR
AcyA_A.flavus_        521 ----DTKPAEEQEG----MYELDTDLSHMEGIVRKRSPQPSADRNQSVDGDSKLHEEGKS
AcyA_A.oryzae_        521 ----DTKPAEEQEG----MYELDTDLSHMEGIVRKRSPQPSADRNQSVDGDSKLHEEGKS
AcyA_A.nidulans_      515 ---KETNPSEAPG-----VWALDTDLSHMEGIVQ----PAADDGDKTNEGKIVRHDEKRL
AcyA_N.crassa_        649 -MDEDIGPTDMHMGGG-TVYHLDTNLNDMEGILTKPQPMTPLDNSISMRRESEKMIVPIT
AcyA_C.albicans_      257 ----------------------------------KEEPHAPKIEEVAENDVAIDNVLEKK
AcyA_S.cerevisiae_    573 -DYDTSSTSSSLPSSSISSEHTSSCSDSSSYTNAYMEANREQDNKTPILNKIKSYTKKFT
consensus             721     . ....        .. ............     .  .  .    . .. .    .. .


AcyA_A.fumigatus_     578 SEGLPPGHWDAPESWQVKKHGEDLAARLPNVASDATGIAREPDGTSYFIRVFRIDSTFAT
AcyA_A.clavatus_      581 QEAAPAGHWDAPESWQVKKHGEDLAARLPSTAGDTGLVVPERDGLSYFIRVFRIDSTFAT
AcyA_A.niger_         464 RDAVPAGNWDAPESWHVKKQ-NEPSTLLPQDTSEPGPTITEPDGPNWYIRIFRPDATFAT
AcyA_A.flavus_        573 RDGLPTGHWDAPDSWHVRRHGEDNAPPFPSGDIEVARTVPQPDGAPYFIRVFRVDSTFAT
AcyA_A.oryzae_        573 RDGLPTGHWDAPDSWHVRRHGEDNAPPFPSGDIEVARTVPQPDGAPYFIRVFRVDSTFAT
AcyA_A.nidulans_      563 GDQLGAGNWDAPESWHVKRQRNEVLAKVPKMTNDAARTIAEPDGVPYFIRVFRIDGTFAT
AcyA_N.crassa_        707 TD--PEGAWAAPDSWAVRDNKKSLAPQVNEDLCSRQPSEEKEKKSNYYIRVFRSDSTWTT
AcyA_C.albicans_      283 RLPVLYGTHQVPH----------------------VTNSKDIKSSHIIRVFKEDNTFTT
AcyA_S.cerevisiae_    632 SSSVNMNSPDGAQSSGLLLQDEKDDEVECQLEHYYKDFSDLDPKRHYAIRIENTDDTFTT
consensus             781  .... .  ....... ...    .. .   ..     .  . ..   ...    ..**.*..*.*..*

                            Ras-associating (RA) domain
AcyA_A.fumigatus_     638 LSAGLNATVADILLMLGRKSFLQDHLNN-YEIVMKRNDLSRQLDHSERPILMQKRLLEQV
AcyA_A.clavatus_      641 LSAGLNATVADILLMLGRKSFLQDHLNN-YEIVMKKHDLSRQLDHSERPILMQKHLLEQV
AcyA_A.niger_         523 LSGGLYATVADLVHGLSRKSFLTEHISN-YELVMQKNDLSRQLDQTEMPILMQKRMLEQV
AcyA_A.flavus_        633 LSTGLNATVADILVILGRKSFLTDHLHN-YEIVMRRNELTRQLDPSEKPILMQKEMLERI
AcyA_A.oryzae_        633 LSTGLNATVADILVILGRKSFLTDHLHN-YEIVMRRNELTRQLDPSEKPILMQKEMLERI
AcyA_A.nidulans_      623 LSNGLHATVADVLLSLGKKSFLTDHLNN-YEIVMRKNDISRQLDPNEQPILMQKKLLEQI
AcyA_N.crassa_        765 LTLPLTATAEDVIMGVAKKTYLPPGAQDSYSLLIKKHDLFRVLNSAEQPLRIQKRLFQQI
AcyA_C.albicans_      320 ILCPLETTTSELLAIVQKKFFLESTTNFQLSVCIG--NCVKVLEDFEKPLKIQMGLLLLS
AcyA_S.cerevisiae_    692 LSCTPATTVEEIIPALKIKFNITAQGNF--QISLKVGKLSKILRPTSKPILIERKLLLLN
consensus             841 .. .. .*..........*......... .............*.  ...*..........


AcyA_A.fumigatus_     697 GYTAKDRIEEIGREDHSYICRFIFLPTKLSGYSSL-DGDPGFSRMQKFSHVDLQGRSLVT
AcyA_A.clavatus_      700 GYTAKDRIEEIGREDHSYICRFIFLPTKLSGYSSL-DGDPGFNKMQKFSHVDLQGRSLVT
AcyA_A.niger_         582 GYTEKDRIEEVGREDHSYILRFTFLPARITGYIID-GNDPLGKAQQKFSHVDLSNRSLVT
AcyA_A.flavus_        692 GYTSKDRIEEVGREDHSYVIRFTFLPNKLNGLTSLQGGDSGFNRNQKFSHVDLSNRCLVT
AcyA_A.oryzae_        692 GYTSKDRIEEVGREDHSYVIRFTFLPNKLNGLTSLQGGDSGFNRNQKFSHVDLSNRCLVT
AcyA_A.nidulans_      682 GYTEKDRIEELGREDHSYILRFTFLPTKLSGYSSL-EGEPGFSKNQKFSHVDLQGRSLVT
AcyA_N.crassa_        825 GYQEKDGIDEIGREDNSYICRWVFLKEKEADMHLL-SPDINFR-NQKLNHVDLSGRNLIT
AcyA_C.albicans_      378 GYTEEDKIRMLGREDLSFVCKFVVENIFLRSLTHD----EEVLLSRNYVDVNISSLNLKN
```

```
AcyA_S.cerevisiae_   750 GYRKSDPLHIMGIEDLSFVFKFLFHPVTPSHFTPE---QEQRIMRSEFVHVDLRNMDLTT
consensus            901 **. .*.....*.**.*.. ...... ........  .. .. .......*... . *..

AcyA_A.fumigatus_    756 IPITLYKKASEIISLNLSRNLSLDVPKDFIQGCINLREIKFIGNEARRLPASFSLASRLT
AcyA_A.clavatus_     759 IPITLYKKASEIISLNLSRNLSLDVPKDFIQGCINLREIKFVGNEALRLPASFSLASRLT
AcyA_A.niger_        641 IPIGLYKKAPEIISLNLSKNLALDVPKDFIQGCINLREIKFIGNEALRLPASFGLASRLT
AcyA_A.flavus_       752 IPISLYSKAAEIISLNLSKNLSLDVPKDFIQGCINLRELKFIGNEAPRLPASFGLASRLT
AcyA_A.oryzae_       752 IPISLYSKAAEIISLNLSKNLSLDVPKDFIQGCINLRELKFIGNEAPRLPASFGLASRLT
AcyA_A.nidulans_     741 IPIALYKKAPEIISLNLSRNLELDVPKDFIQGCINLREIKYIGSEALRLPPSFSLASRLT
AcyA_N.crassa_       883 IPVPLYRKAAEIVSLNLSRNLSLDVPRDFIQACTALRDIKYNNNEAQALPKSFATASKLT
AcyA_C.albicans_     434 VPIIFHQHTYEIEKLNVANNPSIYLPLDFIQGCTSLAYVDFSHNGCSKFPNNLLEAPQLT
AcyA_S.cerevisiae_   807 PPIIFYQHTSEIESLDVSNNANIFLPLEFIESSIKLLSLRMVNIRASKFPSNITKAYKLV
consensus            961 .*. ..... **..*....*.....*..**.....*.......... ..*... .*..*.
```

Leucine-rich repeat domain, L domain-like

```
AcyA_A.fumigatus_    816 YLDVSNNLLEELGHANLDRLHGLVSIKMANNKLSELPSYFGNFQYLRSLNISSNNFQVFP
AcyA_A.clavatus_     819 YLDVSNNLLEDLDHAHLDSLHGLVSIKMANNKLKTLPSYFGNFQYLRNLNISSNSFQTFP
AcyA_A.niger_        701 YLDVSNNFLEDLTHANLDRLHGLVSIKMANNRLTKLPSYFGNFQYLRSLNISSNNFKVFP
AcyA_A.flavus_       812 YLDVSNNYVEQLDNAGLDKLQGLVSIKLSNNKLTSLPSYFGNFAYLRSLNLSSNNFRVFP
AcyA_A.oryzae_       812 YLDVSNNYVEQLDNAGLDKLQGLVSIKLSNNKLTSLPSYFGNFAYLRSLNLSSNNFRVFP
AcyA_A.nidulans_     801 YLDVSNNFLDQLDHANLDRLQGLVSIKLANNRLTKLPDYFGNFKSLRSLNIASNNFQVFP
AcyA_N.crassa_       943 YLDVSNNRLQDLDHSELSKLTGLLKVNLANNCLRSLPPTLGAYKSLRTLNISSNFLDVFP
AcyA_C.albicans_     494 HLNLEMNFLDEIPQR-ISCLSNLTNLKLSSNQLYSLPHSFSTLTNLKQLDLSSNYFDSYP
AcyA_S.cerevisiae_   867 SLELQRNFIRKVPNS-IMKLSNLTILNLQCNELESLPAGFVELKNLQLLDLSSNKFMHYP
consensus           1021 .*....*........ ...* .*.......*.*..**...... .*..*...**.. ..*

AcyA_A.fumigatus_    876 SFLCNLKSLVDLDISFNNIAELP-NIGKLVTLERLWMTNNMLSGPLDETFKDLVNLREID
AcyA_A.clavatus_     879 SFLCNLKSLVDLDISFNNIAELP-NIGKLTTLERLWVTNNVLRGPLDETFKELVSLKEVD
AcyA_A.niger_        761 DLLCGLKSLVDLDISFNNISELP-QIGKLATLERIWMTNNIVRGALDDTFRDLVNLKEID
AcyA_A.flavus_       872 EFLGNLKNLVDLDISFNNIAEVP-CIGRLSTLERLWMTNNVLRGALDESFKDLVNVKEID
AcyA_A.oryzae_       872 EFLGNLKNLVDLDISFNNIAEVP-CIGRLSTLERLWMTNNVLRGALDESFKDLVNVKEID
AcyA_A.nidulans_     861 EFLCNLKSLVDLDISFNNISELP-NIGNLTSLERLWMTNNGFRGPFGESIKDLVNLKEID
AcyA_N.crassa_      1003 SFICELETIVDLDLSFNSINNLPDNLMKLRNLEKFVITNNRLSGPISESVRDLVSLRELD
AcyA_C.albicans_     553 EAVNKLTNLVELNFSYNDLSIIPESIANLINLQKLNLCTNKLSGTLPGYLSQLKALKRLD
AcyA_S.cerevisiae_   926 EVINYCTNLLQIDLSYNKIQSLPQSTKYLVKLAKMNPSHNKLN--FIGDLSEMTDLRTLN
consensus           1081 ..............*.*.. ..* ....* .*.......*.... ...............

AcyA_A.fumigatus_    935 ARFNAITNIDILAQLPRLEQLLIGHNAVSKFKGSFPKLRTLLLDHCPMTQFDIDAPVPT-
AcyA_A.clavatus_     938 ARFNAITNIDTLAQLPRLEQLLIGHNAVSKFRGSFVKLRTLFLDHCPMTQFDIDAPMPT-
AcyA_A.niger_        820 ARFNEITNIDTLCVLPRLEQVAIGHNAISKFKGSFPKLRTLALDHCPMTQFEIDAPIPT-
AcyA_A.flavus_       931 ARFNEITNIDNLSYVPRLEQLHVGHNAISKFKGSFPKLRTLLLDHCPITQFDIDAPMPT-
AcyA_A.oryzae_       931 ARFNEITNIDNLSYVPRLEQLHVGHNAISKFKGSFPKLRTLLLDHCPITQFDIDAPMPT-
AcyA_A.nidulans_     920 ARFNEIVNIDSLTLLPRLEQLLIGHNSVSKFRGSFPKLRNLVLDHCPVTQFDIDAPMPT-
AcyA_N.crassa_      1063 IRYNQISTIDVLSDLPRLEILSADHNQISKFSGSFERLRSLKLNSNPIVKFEVKAPVPI-
AcyA_C.albicans_     613 IRYNYISNVDVLGIIPNLEVAYASKNAISTFSDQMKCLRLLHFDRNPITELKFNTQMQM-
AcyA_S.cerevisiae_   984 LRYNRISSIKTN--ASNLQNLFLTDNRISNFEDTLPKLRALEIQENPITSISFKDFYPKN
consensus           1141 .*.* *.... .  ...*... ...*..*.*......**.* ....*...........

AcyA_A.fumigatus_    994 LTSLNIASAKLVQFRDSLFENLPNLTKLILDKNHFVSMSPHIGKLRRLEHFSMIKNPLAA
AcyA_A.clavatus_     997 LTSLNIASAKLVQFKDSLFEYLPNLSKLVLDKNHFVSMSPHIGKLRRLEHFSMIKNPLAA
AcyA_A.niger_        879 LTSLNIASAKLVQLRDTFFENLPNLTKVILDKNHFVSMSPHIGKLRKLEHFSMIKNPLAA
AcyA_A.flavus_       990 LTVLNIASAKLVQLRDTLFENAPNLTKLVLDKNHFVSMSSHIGKLRKLEHFSIIKNPLDA
AcyA_A.oryzae_       990 LTVLNIASAKLVQLRDTLFENAPNLTKLVLDKNHFVSMSSHIGKLRKLEHFSIIKNPLDA
AcyA_A.nidulans_     979 LTSLNIASAKLVQFRDTLFDSLPNLTKLILDKNHFMSMSPHIGKLRKLEHFSMAKNPLSS
AcyA_N.crassa_      1122 PQILNLSNAQLASIDES-IDNLMNLERLILDSNYFVSLPNQIGNLKKLDHLSMANNHLGE
AcyA_C.albicans_     672 LSVLDLSRAKITAFPAEFVEKVPNIEKLVLDKNHLVSLPNELCQLSKLVSLSVHANNLQS
AcyA_S.cerevisiae_  1042 MTSLTLNKAQLSSIPGELLTKLSFLEKLELNQNNLTRLPQEISKLTKLVFLSVARNKLEY
consensus           1201 ...*....*.....................*..*...... ....*..*...*...*.* .
```

```
AcyA_A.fumigatus_    1054 LPATIGCLTELKYLNLRECNLRRLPAEIWHCARLETLNVSSNVLDSFPKHGSSPPQPPTD
AcyA_A.clavatus_     1057 LPATIGCLAELKYLNLRECNLRRLPPEIWHCARLETLNVSSNVLDSFPKHGSPPPQLPSD
AcyA_A.niger_         939 LPATIGCLTELKYLNLRECNLRKLPPEIWHCLRLETLNVSSNVLEGFPKHGSPQPQVPSE
AcyA_A.flavus_       1050 LPATFGCLTELRYLNLRECNLSRLPPELWNCNKLETLNVSSNVLDSFPKHGTPMPQLPGE
AcyA_A.oryzae_       1050 LPATFGCLTELRYLNLRECNLSRLPPELWNCNKLETLNVSSNVLDSFPKHGTPMPQLPGE
AcyA_A.nidulans_     1039 LPPSIGCLTELKYLNLRECNLRRLPQEIWYCLKLETLNVSSNVLDSFPKHGGPPPQLPGE
AcyA_N.crassa_       1181 LPPEIGCLTELRTLDVHGNNMRKLPNEIWWANKLEHLNASSNILTEFPKPASRAPQAPGE
AcyA_C.albicans_      732 LPANIGDLRFLKYLDLHSNNLKSLPDQIWDLCHLTSLNVASNNLTSFPKAPYSVVKRLSS
AcyA_S.cerevisiae_   1102 IPPELSQLKSLRTLDLHSNNIRDFVDGMEN-LELTSLNISSNAFGNSSLEN---------
consensus            1261 .*.....*..*..*.....*..... ... . .*..**..**........... .... .

AcyA_A.fumigatus_    1114 T--GITPAA--TPGGATTPSYEELGPLQEQDGLETRRPSQTSGGFISSGGSPND--GTYR
AcyA_A.clavatus_     1117 T--GTTPTP--TPGGTIVPSFEDLGPLEEQ---EARRPSQTSGGFFSTGSSPNG--GTFR
AcyA_A.niger_         999 P--IGTPAA--TPGVSTTPSYEELGALEEQ---EARRPSQTSGGVLSTGSSPNG--GAYR
AcyA_A.flavus_       1110 L----VPG---TPGTSTPTNYEDLAADDQE----GRRPSQTSGSVLSTGGSPNGS-GGYR
AcyA_A.oryzae_       1110 L----VPG---TPGTSTPTNYEDLAADDQE----GRRPSQTSGSVLSTGGSPNGS-GGYR
AcyA_A.nidulans_     1099 ATAGTTPAM--TPGGSVTPSYDDLGTVEEQ---ETRRPSQASGSAPS-GSSPGG--GSTR
AcyA_N.crassa_       1241 AS--PSPGAYPFPNANKNGLLSRTPSMDDLNGDASRRPSQASSTLLGVAVSPVPSGPDNR
AcyA_C.albicans_      792 S-----------------------------------------------------------
AcyA_S.cerevisiae_   1152 ------------------------------------------------------------
consensus            1321      ...   ... .    ..... ...      ........  .... ....  . ..

AcyA_A.fumigatus_    1168 KPSVASSLGQSGRKVSAASRSGTEGSP-------SSRKDSNFSQHVASTFAGSLRNLYLA
AcyA_A.clavatus_     1168 KPSVASSLGQGGRKVSTASRSGTEGSP-------SSRKDSNFSQHVATTFGGSLRNLYLA
AcyA_A.niger_        1050 KPSVASSLGQ-GRKVSAASRSLTDGSP-------SSRKDSNFSQHVATTFGGSLRNLYLA
AcyA_A.flavus_       1158 KPSVASSLSQGGRKVSTASRSLNEGSP-------ASRKDSSFSNLSNQTFCGSLRNLYLA
AcyA_A.oryzae_       1158 KPSVASSLSQGGRKVSTASRSLNEGSP-------ASRKDSSFSNLSNQTFCGSLRNLYLA
AcyA_A.nidulans_     1151 KPSVASSLSQGGRKVSTASK-FAEGSP-------SSRKDSNFSQHVATTFGGSLRNLYLA
AcyA_N.crassa_       1299 KSSMVSLYGKGGRKTSVVSRSTTQSSTGVITPSNGPRKDSSLSYRFTHTFSGSLKNLYLA
AcyA_C.albicans_      793 ---------------------------------------LVDVHLESSQTLSLADSLLILILS
AcyA_S.cerevisiae_   1152 ------------------------------------------SFYHNMSYGSKLSKSLMFFIAA
consensus            1381 ........ ....... ....  ....        ..... .... ......**......
```

Leucine-rich repeat domain, L domain-like

```
AcyA_A.fumigatus_    1221 DNRLEDDIFRELSLIPELRVVNLSYNELTELPQGLLKRWPFLTELYLSGNELTSLPSDD-
AcyA_A.clavatus_     1221 DNRLEDDIFRELSLIPELRVVNLSYNELAELPQGLLKRWPFLTELYLSGNELTSLPSDD-
AcyA_A.niger_        1102 DNRLEDDIFRELSLIPELRIVNLSYNELTELPQGLLRRWPLLTELYISGNELTSLPSDD-
AcyA_A.flavus_       1211 DNRLEDDIFRELSLLTELRVVNLSYNELTELPQGLLKRWSNLTELYLSGNELTSLPSDD-
AcyA_A.oryzae_       1211 DNRLEDDIFRELSLLTELRVVNLSYNELTELPQGLLKRWSNLTELYLSGNELTSLPSDD-
AcyA_A.nidulans_     1203 DNRLEDDVFRELSFIPELRIVNLSYNVLNEIPPGLLKRWPLLTELYLSGNELTSLPSDD-
AcyA_N.crassa_       1359 DNQLDDDVFEELKHLPELRVLNLSCNDLSDMPQGTIRSWPQLVELYLSGNELTSLPAEDF
AcyA_C.albicans_      817 DNRLSDDCFDEISFLIALKSLNVSYNDLIEIPQGTLSRLTRLNELYLSGNELTTLPADD-
AcyA_S.cerevisiae_   1174 DNQFDDAMWPLFNCFVNLKVLNLSYNNFSDVSH---MKLESITELYLSGNKLTTLSGDT-
consensus            1441 **...*...........*...*.*.*.............. ..***.***.**.*....

AcyA_A.fumigatus_    1280 LEEGSNLKVLNLNANRFQVLPAELCKVSKLAILDVGSNALKYNVSNWPYDWNWNWNRNLK
AcyA_A.clavatus_     1280 LEEGSNLKILNLNANRFQVLPAELCKVSKLAILDVGSNSLKYNVSNWPYDWNWNWNRNLK
AcyA_A.niger_        1161 LEEGSSLRVLHINANRFQVLPAELCKVSKLSILDVGSNSLKYNVSNWPYDWNWNWNRNLK
AcyA_A.flavus_       1270 LEEGSQLKILNINANRFQVLPAELCKVSKLSILDVGSNSLKYNVSNWPYDWNWNWNRNLK
AcyA_A.oryzae_       1270 LEEGSQLKILNINANRFQVLPAELCKVSKLSILDVGSNSLKYNVSNWPYDWNWNWNRNLK
AcyA_A.nidulans_     1262 LEEGSSLKILNINANRFQVLPAELCKVSKLSILDVGSNYLKYNVSNWPYDWNWNWNRNLK
AcyA_N.crassa_       1419 LEEHCLLQTLHINGNKFINLPAEISRAKKLQVLDCSSNNLKYNVTNVPYDWNWNFNRDLR
AcyA_C.albicans_      876 LEVLKSLKLLYMNNNKLVSLPAELSRIANLQHLDVSSNQLKYNISNWPYDWSWHWNKNLK
AcyA_S.cerevisiae_   1230 VLKWSSLKTLMLNSNQMLSLPAELSNLSQLSVFDVGANQLKYNISNYHYDWNWRNNKELK
consensus            1501 ..... *..*..*.*....****......*...*...* ****..*..***.*..*..*.
```

```
AcyA_A.fumigatus_    1340 YLNFSGNKRLEIKP---NITSAGTTTANGTDLTDFNSLTHLRVLGLMDVTLTTPTIPEEN
AcyA_A.clavatus_     1340 YLNFSGNKRLEIKP---NITSMGPSATNGADLTDFNSLTHLRVLGLMDVTLTTPTIPEEN
AcyA_A.niger_        1221 YLNFSGNKRLEIKPPGSSLSNQQQQPKHQTNLTDFTSLTHLRVLGLMDVTLTITTIPEEN
AcyA_A.flavus_       1330 YLNFSGNKRLEIKP--HVSSLLGPPNTNGTDLTDFNSLTYLRVLGLMDVTLTTSTIPEEN
AcyA_A.oryzae_       1330 YLNFSGNKRLEIKP--HVSSLLGPPNTNGTDLTDFNSLTYLRVLGLMDVTLTTSTIPEEN
AcyA_A.nidulans_     1322 YLNFSGNKRLEIKP---NVSSLGSQPPNGADLTDFNSLTHLRVLGLMDVTLTTSNIPEEN
AcyA_N.crassa_       1479 YLNLSGNKRLEIKN---NYRQPQSYRDDDFADTDFSKLTNLRVLGLMEVTHTLPNIPDQT
AcyA_C.albicans_      936 YLNFSGNKRFEIKQ------SHIKNPETGEDFDSLLVLKQLRVLGLIDVTLTTINVPEQA
AcyA_S.cerevisiae_   1290 YLNFSGNRRFEIKS--------FISHDIDADLSDLTVLPQLKVLGLMDVTLNTTKVPDEN
consensus            1561 ***.***.*.***.     .  .    ..  ......*.  *.****..**......*...


AcyA_A.fumigatus_    1397 EDRRVRTSASLAG-SLAYGMADFLGKSEHLSIIDMIVPRLKPDNVETLVGLFDGQAQST-
AcyA_A.clavatus_     1397 EDRRVRTSASLAG-SLAYGMADFLGRSEHLSIIDMIVPRLKPDNVETLVGLFDGQAQST-
AcyA_A.niger_        1281 EDRRVRTSASLAG-SLAYGMADFLGKSEHLSIFDMIVPRLKPDKVETVVGMFDGQSQSS-
AcyA_A.flavus_       1388 EDRRVRTSASLAG-FLAYGMADFLGKSEHLSIIDMIVPRLKPDNVETVVGMFDGTSQST-
AcyA_A.oryzae_       1388 EDRRVRTSASLAG-FLAYGMADFLGKSEHLSIIDMIVPRLKPDNVETVVGMFDGTSQST-
AcyA_A.nidulans_     1379 EDRRVRTSASLAG-SLAYGMADFLGRSEHLSIIDMIVPRMRQDNVETVVGMFDGQPSST-
AcyA_N.crassa_       1536 EDRRVRTSEMKAGAYLPYGMADTLGKNEHLSLFDLVVPRLGSVETDTLVALFDGKELST-
AcyA_C.albicans_      990 VDLRLRTTASEFD-NFGYGVSDSLGMRDHVSARDLFVQKFRGKENEMLLCAFDGKHGATN
AcyA_S.cerevisiae_   1342 VNFRLRTTASIIN-GMRYGVADTLGQRDYVSSRDVTFERFRGNDDECSLCLHDSKNQNAD
consensus            1621 ...*.**......  ..**..*.**.....*..*................*.  ...


AcyA_A.fumigatus_    1455 GGSRIAKFLHENFTSTFSSELKRLRVEEEETPLDALRRIFLALNKNMAAAAYKSIDDRSV
AcyA_A.clavatus_     1455 GGSRIAKFLHENFTSTFSSELKRLRLDEQETPLDALRRIFLVLNKNMAAAAYRSIDDRSV
AcyA_A.niger_        1339 GGSRIAKFLHENFIHTFSSELKRMRPEQHETPLDALRRAFLTLNKNMAFACYKSLD-QDV
AcyA_A.flavus_       1446 GGSKIAKYLHENFLRTFSTELRKLRRDLQETPLDALRRAFLALNKNMAAACFKTFDDKTV
AcyA_A.oryzae_       1446 GGSKIAKYLHENFLRTFSTELRKLRRDLQETPLDALRRAFLALNKNMAAACFKTFDDKTV
AcyA_A.nidulans_     1437 GGSRVAKFLHENFLHTFSAELKRLRRDEQETPLDAFRRIFLTLNRNMAFACYKSID-QDV
AcyA_N.crassa_       1595 GGSKIAKFLYEEFSRLFILELEKVKGKPNENPADALRRIFLSVNK-LLMSLSNSADERGL
AcyA_C.albicans_     1049 QGHRISLVAKNMFVRNFTKELDEIKSD--DEIENALRKAFLNFNKEINGILTAKKN-KSF
AcyA_S.cerevisiae_   1401 YGHNISRIVRDIYDKILIRQLERYGDETDDNIKTALRFSFLQLNKEINGMLNSVDN----
consensus            1681 .*..................*.... . ......*.*..** .*...... ... .....


                                  PPM-type phosphatase domain
AcyA_A.fumigatus_    1515 RQFHRGSPATK--LLNQDDIRSGGVATVLYLNNMDLYVANVGDAQAILVKSDGTLKYLTK
AcyA_A.clavatus_     1515 RQFHRGSPATK--LLNQDDIRSGGVATVLYLNNMDLYVANVGDAQAILVKSDGSLRHLTK
AcyA_A.niger_        1398 RQYQEDSTDQRKVRLTKDDIDLGGVATVMYLNNMDLYVANVGDAQAILVKADGSLRHLTQ
AcyA_A.flavus_       1506 QRYEE-PPDQKSRKLTNEDGASGGVATVLYLRNMDLFVANVGDAQAILVKSDGSLRHLTR
AcyA_A.oryzae_       1506 QRYEE-PPDQKSRKLTNEDGASGGVATVLYLRNMDLFVANVGDAQAILVKSDGSLRHLTR
AcyA_A.nidulans_     1496 RLFQEDSSDQKKVRLNKEDLQSGGVATVLYLNNTDLYAANIGDAQAILVKSDGSMRYLTR
AcyA_N.crassa_       1654 DSHPSRNYAHT--VLTKEDLNSGCVATVAYLSELKLYVANVGDVQGMLIQANGSFKMLTK
AcyA_C.albicans_     1106 TPVPNMSKEALELNLVDDG-NAGCTVSVIYIKDKKLYSANIGDIEALLCRNNGDQFLLTE
AcyA_S.cerevisiae_   1457 --------GADVANLSYADLLSGACSTVIYIRGKKLFAANLGDCMAILSKNNGDYQTLTK
consensus            1741   .   . . .     *. ... .*....*.*.  ...*..**.**....*....*...  **.


AcyA_A.fumigatus_    1573 NHDPAEPHERARIRAAGGFVSRNGRLNDILHVSRAFGHFQLMPAVVAAPHTMHVQLTEQD
AcyA_A.clavatus_     1573 NHDPAEPHERARIRAAGGFVSRNGRLNDILHVSRAFGHFQLMPAVIAAPHTMHVNLTEQD
AcyA_A.niger_        1458 NHDPAETHERARIRDAGGFVSRTGKLNDVLTVSRAFGHFPMMPAVIAAPHTMHVNLTEQD
AcyA_A.flavus_       1565 THDPAEPHERARIRAAGGYVSRQGKLNDVLPVSRSFGHFHLMPALIAAPHTMHVNLTEQD
AcyA_A.oryzae_       1565 THDPAEPHERARIRAAGGYVSRQGKLNDVLPVSRSFGHFHLMPALIAAPHTMHVNLTEQD
AcyA_A.nidulans_     1556 NHDPAEAGERARIRAAGGFVSRNGRLNDYLPVSRSFGYFNLMPAVIAAPHTMHVSLTEQD
AcyA_N.crassa_       1712 KHDPADPVERSRIRNAGGWVSRNGRLNDVLNVSRAFGYTELLPAVQAAPDITEHTIDDKD
AcyA_C.albicans_     1165 KHDPTNREEFERIRASGGYVSGGGELDGQLSVSRGVGFFNFLPHTHCGPTIRRFKITNDD
AcyA_S.cerevisiae_   1509 QHLPTKREEYERIRISGGYVN-NGKLDGVVDVSRAVGFFDLLPHIHASPDISVVTLTKSD
consensus            1801 .*.*....*..***..**.*...*.*....  ***..*..  ..*.....*..........*


AcyA_A.fumigatus_    1633 EMIILASKELWDYVTPDVVVDVTRAERRDLMIAAQKIRDLAISFGANNKLMVMILGVSDL
AcyA_A.clavatus_     1633 EMIILASKELWDYVTPDVVVDVTRAERRDLMIAAQKIRDLAISFGANNKLMVMILGVSEL
```

```
AcyA_A.niger_        1518 EMVIIASRELWDYVTPDLVVDVTRAERRDLMIAAQKIRDLAISFGATNKLMVMILGVGDL
AcyA_A.flavus_       1625 EMIILASRELWDYVTPDLVVDVTRAERRDLMVAAQKIRDLAISFGANNKLMVMILGVGDL
AcyA_A.oryzae_       1625 EMIILASRELWDYVTPDLVVDVTRAERRDLMVAAQKIRDLAISFGANNKLMVMILGVGDL
AcyA_A.nidulans_     1616 EMIILASKELWDYVTPDLVVDVTRAERRDLMVAAQKIRDLALSFGANNKLMVMILGVGDL
AcyA_N.crassa_       1772 ETVLIASKELWEHLRPELIVDVARECRSDLMKASQKLRDLAIAYGSTNKLLIMMIGVANL
AcyA_C.albicans_     1225 DMIILGSKQLWDFISYESAVDIIRKDKNDPMVAAQKLRDFAICYGATDKICVIVLTFGNR
AcyA_S.cerevisiae_   1568 EMLIVATHKLWEYMDVDTVCDIARENSTDPLRAAAELKDHAMAYGCTENITILCLALYEN
consensus            1861 .........**.........*..*....*...*.....*.*...*...............
```

```
AcyA_A.fumigatus_    1693 KKREK-FKFRGTSLSMGPPAFPEEQIIPS-TKRTKKPRDMPGDSRLARF-DYVDAPIGEL
AcyA_A.clavatus_     1693 KKREK-FKFRGTSLSMGPSAFPEGEIIPS-TKRTKKPRDAPGDSRLARF-DYVDAPIGEL
AcyA_A.niger_        1578 KRREK-IRTRPSLSMVGP---PEEQIIPS-TKRTKKPRDAPGDSRLARF-NYVDAPVGEL
AcyA_A.flavus_       1685 KKRDRQSKFRNASVSTGMLE--EQQILPT-SKRTKKPRDMPGDSRLARF-EFVDAPVGEL
AcyA_A.oryzae_       1685 KKRDRQSKFRNASVSTGMLE--EQQILPT-SKRTKKPRDMPGDSRLARF-EFVDAPVGEL
AcyA_A.nidulans_     1676 RKRER---RPPRFPSMNSFSQVDDSILPS-PKRTKKPRDMPGDSRLARF-DYVDAPTGEL
AcyA_N.crassa_       1832 KQRQAQQFKGQLNATFSMPQDDPSHVPPSGNKRRKVRAEGPLDSNLMRLNAEVPPPTGQL
AcyA_C.albicans_     1285 QKQAANMYSNYGVD----------------RRRRDKQQVVGGDSNLRKLEQEIEPPIGPL
AcyA_S.cerevisiae_   1628 IQQQN--------------------RFTLNKNSLMTRRSTFEDITLRRLQPEISPPTGNL
consensus            1921 .....  . .    . .     .  .... ............*..*... .....*.*.*
```

Nucleotide cyclase

```
AcyA_A.fumigatus_    1750 AIIFTDIKKSTSLWETCPDAMRSAIQIHNDILRRQLGIIGGYEVKTEGDAFMVAFSTTTA
AcyA_A.clavatus_     1750 AIIFTDIKKSTSLWETCPDAMRSAIQIHNDILRRQLGIIGGYEVKTEGDAFMVAFSTTTA
AcyA_A.niger_        1632 AIIFTDIKKSTSLWETCPDAMRSAIQIHNDILRRQLGIIGGYEVKTEGDAFMVAFSTTTA
AcyA_A.flavus_       1741 AIIFTDIKGSTSLWETCPDAMRSAIQIHNDILRRQLGIIGGYEVKTEGDAFMVAFSTTTA
AcyA_A.oryzae_       1741 AIIFTDIKGSTSLWETCPDAMRSAIQIHNDILRRQLGIIGGYEVKTEGDAFMVAFSTTTA
AcyA_A.nidulans_     1731 AIIFTDIKQSTGLWETCPDAMRSAIQIHNDILRRQLGIIGGYEVKTEGDAFMVAFSTTTA
AcyA_N.crassa_       1892 SIVFTDIKNSTQLWENYPEAMRLAIKLHNEVMRRQLRMIGGFEVKTEGDAFMVSFPTATS
AcyA_C.albicans_     1329 ALVFTDIKNSTLLWDSYPAPMRSAIKIHNTIMRRQLRITGGYEVKTEGDAFMVAFPSPTA
AcyA_S.cerevisiae_   1668 AMVFTDIKSSTFLWELFPNAMRTAIKTHNDIMRRQLRIYGGYEVKTEGDAFMVAFPTPTS
consensus            1981 ...***** **.**...*..**.**..**...****...**.***********.*...*.
```

```
AcyA_A.fumigatus_    1810 ALLWCFNCQTQLLEAEWPTEILEQPQCQVQYDMENNIIFRGLSVRMGIHWGEPVCEKDPV
AcyA_A.clavatus_     1810 ALLWCFNCQTQLLEAEWPTEILEQPQCQVQYDMDNNIIFRGLSVRMGVHWGEPVCEKDPV
AcyA_A.niger_        1692 ALLWCFNCQTQLLDAEWPTEILEQPQCQVVYDTENNIIFRGLSVRMGSHWGEPVCEKDPV
AcyA_A.flavus_       1801 ALLWCFNCQMQLLEAEWPTEILEQTQCQVEYDMDNNIIFRGLSVRMGIHWGEPVCEKDPV
AcyA_A.oryzae_       1801 ALLWCFNCQMQLLEAEWPTEILEQTQCQVEYDMDNNIIFRGLSVRMGIHWGEPVCEKDPV
AcyA_A.nidulans_     1791 ALLWCFNCQYQLLEAEWPTEILEQPQCQVQFDMENNIIFRGLSVRMGIHWGEPVCEKDPI
AcyA_N.crassa_       1952 ALLWCFAVQMKLLTVDWPPEVLSNSSCQPIYDRNNNLITRGLSVRMGAHWGEPLAERDPV
AcyA_C.albicans_     1389 ALLWCFQVQQNLVTADWPSEILETDQCCVVSDSENNTIFRGLSVRMGIHWGSPVCEPDVI
AcyA_S.cerevisiae_   1728 GLTWCLSGQLKLLDAQWPEEITSVQDGCQVTDRNGNIIYQGLSVRMGIHWGCPVPELDLV
consensus            2041 .*.**...* .*....**.*.... .... .*...*.*..*******.***.*..*.*..
```

```
AcyA_A.fumigatus_    1870 TNRMDYFGPMVNRASRISAVADGGQIFVSSDFMSDIQRNLEVYADSERAASTGSEESYAV
AcyA_A.clavatus_     1870 TNRMDYFGPMVNRASRISAVADGGQIFVSSDFMSDIQRNLEVYADSERAASTGSEESYAV
AcyA_A.niger_        1752 TNRMDYFGPMVNRASRISAVADGGQIFVSSDFMSDIQRNLEIFADSERAASTGSEESYAY
AcyA_A.flavus_       1861 TNRMDYFGPMVNRASRISAVADGGQIFVSSDFMSDIQRNLEVFADSERAASTGSEEGYAV
AcyA_A.oryzae_       1861 TNRMDYFGPMVNRASRISAVADGGQIFVSSDFMSDIQRNLEVFADSERAASTGSEEGYAV
AcyA_A.nidulans_     1851 TNRMDYFGPMVNRASRISAVADGGQIFVSSDFMNDMQRNLELFADSERAASTGSEESYAL
AcyA_N.crassa_       2012 TRRMDYYGPMVNKASRISAVADGGQITASSDFITEIHRCLETYKESVDVD----EDSLED
AcyA_C.albicans_     1449 TGRMDYFGPMVNRASRISAIADGGQIAVSSDFLDVLNSLTVKHNNIKNNVESLIDAYQGN
AcyA_S.cerevisiae_   1788 TQRMDYLGPMVNKAARVQGVADGGQIAMSSDFYSEFN-KIMKYHERVVKGKESLKEVYGE
consensus            2101 *.****.*****.*.*....******..****............................
```

Nucleotide cyclase

```
AcyA_A.fumigatus_    1930 DSLGYNIRRELQQLNSQGFVIKDQGERKLKGLENPEPLYLIYPHSLSGR--------LTT
AcyA_A.clavatus_     1930 DSLGYNIRRELQQLNSQGFVIKDQGERKLKGLENPEPLYLIYPHSLSGR--------LTT
AcyA_A.niger_        1812 DTLGHNIRRELQQLNSQGFVIKDQGERKLKGLENPEPLYLIYPHGLSGR--------LTT
AcyA_A.flavus_       1921 DNLGYNIRRELQQLNSLGFVIKDQGERKLKGLENPEPLYLIYPHTLSGR--------LSI
AcyA_A.oryzae_       1921 DNLGYNIRRELQQLNSLGFVIKDQGERKLKGLENPEPLYLIYPHTLSGR--------LSI
```

```
AcyA_A.nidulans_     1911 D-LGDNIRRELQQLNSQGFVIKDQGERKLKGLENPEPLYLIYPHALSGR--------LST
AcyA_N.crassa_       2068 DATAKAIRAELRALSSQGFEVKDMGEKKLKGLENPELVYSVYPHALVGRTEQHHAHTEST
AcyA_C.albicans_     1509 ENAGMTIERELNALEDLGCNYFKIGERKLKGLETPEPITLVFTNRLKLR--------YDI
AcyA_S.cerevisiae_   1847 EIIGEVLEREIAMLESIGWAFFDFGEHKLKGLETKELVTIAYPKILASR-----------
consensus            2161 . .. ....*...*...*......**.******..*........ *..*        ...


AcyA_A.fumigatus_    1982 LEQAEARE------NGPTTISKHSELEIQADLIWRLWELTLRLERLCGALENPGEARLKE
AcyA_A.clavatus_     1982 LEQAESTE------NGPTTISKHSQLEIQADLIWRLWEITLRLERLCGALENPGDARLTE
AcyA_A.niger_        1864 LDKDASNE------LAPTTISKHSQLEIQTDLIWRLWEITLRLERLCAALEYPGEARLKE
AcyA_A.flavus_       1973 MQKDAASDK-----NVPTTISKHSQLEIQTDLIWRLWEITLRLERLCGALEYPGEARLKE
AcyA_A.oryzae_       1973 MQKDAASDK-----NVPTTISKHSQLEIQTDLIWRLWEITLRLERLCGALEYPGEARLKE
AcyA_A.nidulans_     1962 QDQMSGEE------STPTTISQHSQLQIQTEAIWRLWEITLRLERLCGALEHPGEPRLDK
AcyA_N.crassa_       2128 QNQAALQPYGALATPKPATMDPDSEIQFEPETIWALWRVALRLEMLCSTLEEPNARGLQA
AcyA_C.albicans_     1561 FQKRLDAN-----------HSTRVAGTLPVEIIYGLRTVSLRLENLCSSINNGGNYCSEG
AcyA_S.cerevisiae_   1896 -HEFASED--------------EQSKLINETMLFRLRVISNRLESIMSALSGGFIELDSR
consensus            2221 .   .  .        ............. .....*.....***....... ...... .


AcyA_A.fumigatus_    2036 PNVALFNLVKNHGG----ELADSTVVSLVEQQVTRIEMNVTTLSVRYMLKPFKPGDKLSD
AcyA_A.clavatus_     2036 PNLALFNLVKNQGG----ELADSTVVSLVEQQVTRIEMAINTLAVRHMMRPFKPGDKLSD
AcyA_A.niger_        1918 PNVALFNVVKNHGG----ELADSTVISLVEQQVTRIEVCITTLFIRNMMRPFRPGDQLND
AcyA_A.flavus_       2028 PNVSLFNVVKNHGG----ELADSTVVSLVEQQVTRIEMTVNTLAIRNMLRPFRPHDRLND
AcyA_A.oryzae_       2028 PNVSLFNVVKNHGG----ELADSTVVSLVEQQVTRIEMTVNTLAIRNMLRPFRPHDRLND
AcyA_A.nidulans_     2016 PNSALFDIIKKHGG----ELADSSVVSLIEQQVTRIEVAISTLALRHMLRPFKPGDRLDD
AcyA_N.crassa_       2188 PETELLERMKQRAG----EVTDHFLLNFMEHQISRIETCITSLAVRHIAIGSGPITKLND
AcyA_C.albicans_     1610 FESSSGVISQKMNS----SFKDSDLISLLNHVTTRIESCTTTLFLRQQLSQIKGNGGLIE
AcyA_S.cerevisiae_   1941 TEGSYIKFNPKVENGIMQSISEKDALLFFDHVITRIESSVALLHLRQQRCS--GLEIFRN
consensus            2281 ... ..........    ................***. ...*..*........ ... .


AcyA_A.fumigatus_    2092 HAVPIGVVMQQLQTQLAEYRALKEQLAVGGAGITGVSSSSPGAEGQDTGESDPSSASSSF
AcyA_A.clavatus_     2092 HAAPIDEVMQQLQTQLAEYRALKEQLAVSAAGITGTPSTSKGSGAQVTSGSSPSSASSSF
AcyA_A.niger_        1974 HAVPITDVMQQLQAQLAEFRALKEQIDASAGTATSYTYTDPQYS---NGESNYDSASSSY
AcyA_A.flavus_       2084 HAMPIGDVLQELQTQLAEYRALKEQISVSAAGITGSSPSYTASL-----EDSNSSVSSSF
AcyA_A.oryzae_       2084 HAMPIGDVLQELQTQLAEYRALKEQISVSAAGITGSSPSYTASL-----EDSNSSVSSSF
AcyA_A.nidulans_     2072 HAAPIGDVLQELRTQLAEYRALKEQIATNGAGITGASPSSTATDLHYTPDFHSSASSSSF
AcyA_N.crassa_       2244 LRAPMNDVLSTLKEQMDELARYKAKYGSLDQAETDDATDNNSSG-----DVDTLDGSDTE
AcyA_C.albicans_     1666 TNN--SPSLDVIMDEVADIMKTVNELK---------------------------------
AcyA_S.cerevisiae_   1999 DKTSARSNIFNVVDELLQMVKNAKDLST--------------------------------
consensus            2341 .. .. .... ...............   ...... . .  ..      .   .. ....


AcyA_A.fumigatus_    2152 LQLATSSE---------
AcyA_A.clavatus_     2152 LQLAPPSE---------
AcyA_A.niger_        2031 LHMGPPSDEYNPGGPRP
AcyA_A.flavus_       2139 LRMSIP-----------
AcyA_A.oryzae_       2139 LRMSIP-----------
AcyA_A.nidulans_     2132 T----------------
AcyA_N.crassa_       2299 QE---------------
AcyA_C.albicans_          -----------------
AcyA_S.cerevisiae_        -----------------
consensus            2401 . .
```

Figure S1. Alignment of amino acid sequences of AcyA and its putative orthologs of different fungi. Identical and similar residues are shaded in black and gray, respectively.

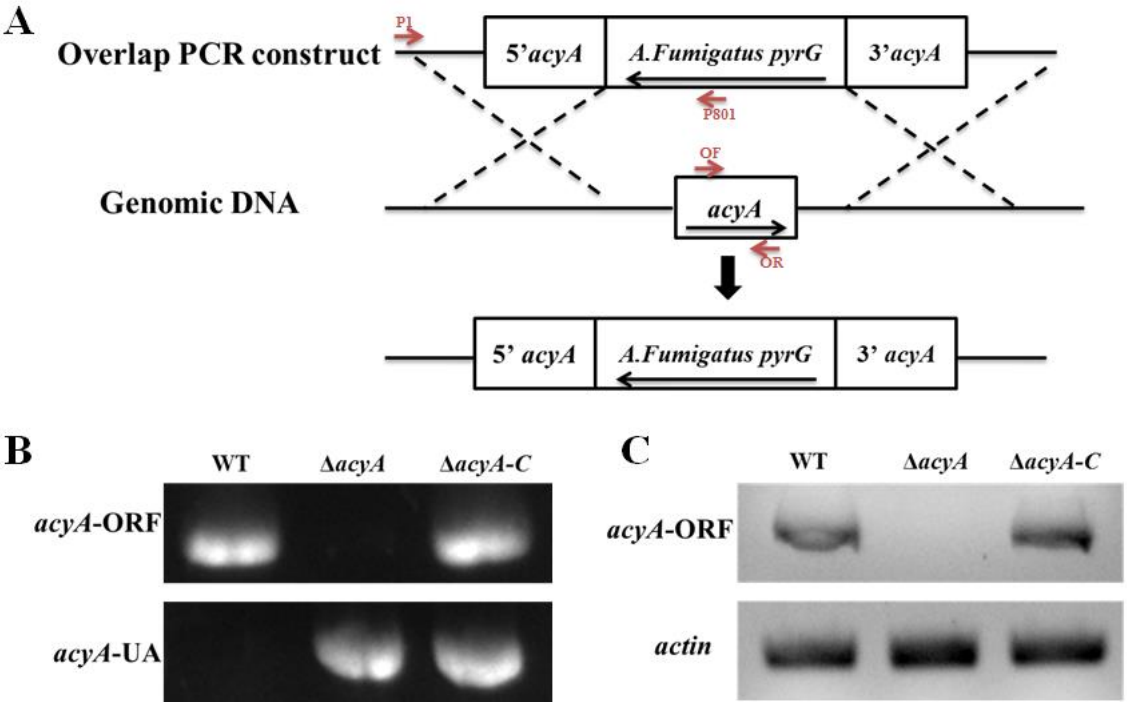

Figure S2. Deletion of *acyA* gene from *A. flavus*. (A) The gene replacement strategy of ***acyA***. **(B)** Diagnostic PCR were performed to confirm the gene deletion and complemented strains, *acyA* ORF was confirmed by primers *acyA*/OF and *acyA*/OR, UA was confirmed by primers *acyA*/p1 and P801.**(C)** RT-PCR verification of *acyA* gene deletion. Actin gene was used as a reference.

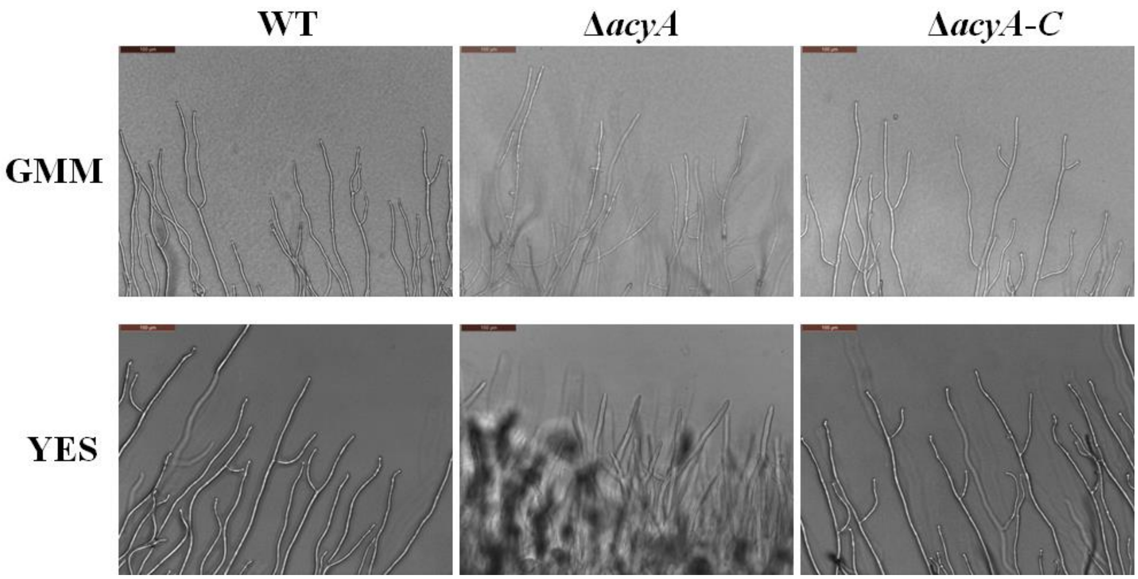

Figure S3. Microscopic examination revealed the difference mycelia tips of WT, Δ*acyA* and Δ*acyA-C* complemented strain when growing in GMM and YES medium, bars=100 μm.
